# Genomic analysis of *Salmonella* isolated from surface water and animal sources in Chile reveals new T6SS effector protein candidates

**DOI:** 10.3389/fmicb.2024.1496223

**Published:** 2024-12-11

**Authors:** Fernando A. Amaya, Carlos J. Blondel, Felipe Reyes-Méndez, Dácil Rivera, Andrea Moreno-Switt, Magaly Toro, Consuelo Badilla, Carlos A. Santiviago, David Pezoa

**Affiliations:** ^1^Laboratorio de Microbiología, Departamento de Bioquímica y Biología Molecular, Facultad de Ciencias Químicas y Farmacéuticas, Universidad de Chile, Santiago, Chile; ^2^Facultad de Medicina y Facultad de Ciencias de la Vida, Instituto de Ciencias Biomédicas, Universidad Andrés Bello, Santiago, Chile; ^3^Núcleo de Investigación en One Health, Facultad de Medicina Veterinaria y Agronomía, Universidad de Las Américas, Santiago, Chile; ^4^Escuela de Medicina Veterinaria, Facultad de Agronomía e Ingeniería Forestal, Facultad de Ciencias Biológicas y Facultad de Medicina, Pontificia Universidad Católica de Chile, Santiago, Chile; ^5^Joint Institute for Food Safety and Applied Nutrition (JIFSAN), University of Maryland, College Park, MD, United States; ^6^Instituto de Nutrición y Tecnología de los Alimentos (INTA), Universidad de Chile, Santiago, Chile; ^7^Departamento de Ciencias Químicas y Biológicas, Universidad Bernardo O'Higgins, Santiago, Chile

**Keywords:** *Salmonella*, T6SS, Chile, effector, immunity protein

## Abstract

Type VI Secretion Systems (T6SS), widely distributed in Gram-negative bacteria, contribute to interbacterial competition and pathogenesis through the translocation of effector proteins to target cells. *Salmonella* harbor 5 pathogenicity islands encoding T6SS (SPI-6, SPI-19, SPI-20, SPI-21 and SPI-22), in which a limited number of effector proteins have been identified. Previous analyses by our group focused on the identification of candidate T6SS effectors and cognate immunity proteins in *Salmonella* genomes deposited in public databases. In this study, the analysis was centered on *Salmonella* isolates obtained from environmental sources in Chile. To this end, bioinformatics and comparative genomics analyses were performed using 695 genomes of *Salmonella* isolates representing 44 serotypes obtained from surface water and animal sources in Chile to identify new T6SS effector proteins. First, T6SS gene clusters were identified using the SecreT6 server. This analysis revealed that most isolates carry the SPI-6 T6SS gene cluster, whereas the SPI-19 and SPI-21 T6SS gene clusters were detected in isolates from a limited number of serotypes. In contrast, the SPI-20 and SPI-22 T6SS gene clusters were not detected. Subsequently, each ORF in the T6SS gene clusters identified was analyzed using bioinformatics tools for effector prediction, identification of immunity proteins and functional biochemical prediction. This analysis detected 20 of the 37 T6SS effector proteins previously reported in *Salmonella*. In addition, 4 new effector proteins with potential antibacterial activity were identified in SPI-6: 2 Rhs effectors with potential DNase activity (PAAR-RhsA-NucA_B and PAAR-RhsA-GH-E) and 2 effectors with potential RNase activity (PAAR-RhsA-CdiA and RhsA-CdiA). Interestingly, the repertoire of SPI-6 T6SS effectors varies among isolates of the same serotype. In SPI-19, no new effector protein was detected. Of note, some Rhs effectors of SPI-19 and SPI-6 present C-terminal ends with unknown function. The presence of cognate immunity proteins carrying domains present in *bona fide* immunity proteins suggests that these effectors have antibacterial activity. Finally, two new effectors were identified in SPI-21: one with potential peptidoglycan hydrolase activity and another with potential membrane pore-forming activity. Altogether, our work broadens the repertoire of *Salmonella* T6SS effector proteins and provides evidence that SPI-6, SPI-19 and SPI-21 T6SS gene clusters harbor a vast array of antibacterial effectors.

## Introduction

The type VI secretion system (T6SS) is an apparatus composed of 13 structural proteins and several accessory proteins that deliver protein effectors into target cells by means of a contractile mechanism (Coulthurst, 2019; Cherrak et al., 2019). The T6SS needle is composed of an inner tube made of a stack of Hcp hexamer rings that is tipped by a trimer of VgrG and a proline-alanine–alanine-arginine repeat (PAAR) protein. This internal structure is surrounded by a contractile sheath of polymerized TssB/TssC subunits assembled in an extended, metastable conformation (Silverman et al., 2013; Cherrak et al., 2019). Contraction of the sheath propels the needle complex toward the target cell (Brackmann et al., 2017). T6SS effector proteins are classified as either cargo or specialized effectors. Cargo effectors are transported by non-covalent interaction with some core components (Coulthurst, 2019), while specialized effectors are VgrG, Hcp or PAAR proteins carrying additional domains (Durand et al., 2014; Whitney et al., 2014; Diniz and Coulthurst, 2015; Ma et al., 2017; Pissaridou et al., 2018).

T6SS effector proteins can target prokaryotic and/or eukaryotic cells (Coulthurst, 2019; Monjarás Feria and Valvano, 2020). Among the anti-bacterial effector proteins, some target the peptidic or glycosidic bonds of the peptidoglycan (Ma and Mekalanos, 2010; Russell et al., 2012; Srikannathasan et al., 2013; Whitney et al., 2013; Berni et al., 2019; Wood et al., 2019), or the FtsZ cell division ring (Ting et al., 2018). These anti-bacterial effectors are usually encoded in bi-cistronic elements with their cognate immunity proteins (E/I pairs) in order to avoid self-intoxication and killing of sibling cells (Russell et al., 2012). Other T6SS effectors target eukaryotic cells, such as those disrupting the actin or microtubule cytoskeleton networks (Monjarás Feria and Valvano, 2020), while trans-kingdom effectors target both bacterial and eukaryotic cells (Jiang et al., 2014). These effectors include those forming pores in membranes or targeting conserved molecules such as NAD^+^ and NADP^+^, and macromolecules such as DNA, RNA and phospholipids (Whitney et al., 2015; Tang et al., 2018; Ahmad et al., 2019). In many enteric pathogens (e.g., *Salmonella*, *Shigella* and *Vibrio*), the T6SS contributes to colonization of the intestinal tract of infected hosts (Sana et al., 2016; Chassaing and Cascales, 2018). On the other hand, strains of the gut commensal *Bacteroides fragilis* use their T6SSs for competition against other Bacteroidales species (Coyne and Comstock, 2019). Hence, the T6SS is a key player in bacterial warfare.

The *Salmonella* genus includes more than 2,600 serotypes distributed between species *S. enterica* and *S. bongori* (Issenhuth-Jeanjean et al., 2014), which differ in clinical signs and host range (Uzzau et al., 2000). In *Salmonella*, five T6SS gene clusters have been identified within *Salmonella* Pathogenicity Islands (SPIs) SPI-6, SPI-19, SPI-20, SPI-21, and SPI-22 (Blondel et al., 2009; Fookes et al., 2011; Bao et al., 2019). These T6SS gene clusters are distributed in 4 different evolutionary lineages: The SPI-6 T6SS gene cluster belongs to subtype i3, SPI-19 T6SS gene cluster to subtype i1, SPI-22 T6SS gene cluster to subtype i4a, and both SPI-20 and SPI-21 T6SS gene clusters to subtype i2 (Bao et al., 2019). Besides their distinct evolutionary origin, these five T6SS gene clusters are differentially distributed among distinct serotypes, subspecies, and species of *Salmonella* (Blondel et al., 2009; Bao et al., 2019).

In *Salmonella*, only a few studies have addressed the role played by the T6SSs in interbacterial and eukaryotic relationships, and most of our understanding regarding the contribution of T6SSs to *Salmonella* infection cycle, virulence and pathogenesis comes from studies of T6SS_SPI-6_ in *S.* Typhimurium and T6SS_SPI-19_ in *S.* Dublin (Mulder et al., 2012; Pezoa et al., 2013; Pezoa et al., 2014; Sana et al., 2016; Sibinelli-Sousa et al., 2022; Xian et al., 2020; Blondel et al., 2010; Hespanhol et al., 2022). Furthermore, knowledge of the presence and distribution of T6SS effector proteins is derived from studies using strains representing a limited number of serotypes (Russell et al., 2012; Benz et al., 2013; Sana et al., 2016; Whitney et al., 2013; Sibinelli-Sousa et al., 2020; Lorente-Cobo et al., 2022; Koskiniemi et al., 2014; Amaya et al., 2022; Jurėnas et al., 2022; Blondel et al., 2023). Consequently, information regarding *Salmonella* T6SS effector proteins is still scarce. Indeed, only 37 T6SS effectors and candidate effectors that target different bacterial molecules such as peptidoglycan, nucleic acids and bacterial ribosomes have been currently identified in a few serotypes (Blondel et al., 2009; Russell et al., 2012; Benz et al., 2013; Whitney et al., 2013; Koskiniemi et al., 2014; Sana et al., 2016; Ho et al., 2017; Sibinelli-Sousa et al., 2020; Amaya et al., 2022; Jurėnas et al., 2022; Lorente-Cobo et al., 2022; Hespanhol et al., 2022; Blondel et al., 2023). This is an important knowledge gap as the T6SS effector proteins are the ultimate mediators of the T6SS activity and thus, their identification and characterization are pivotal for a better understanding of *Salmonella* infectious cycle and in its contribution to environmental fitness and pathogenic potential.

Nowadays, there is increasing evidence that *Salmonella enterica* can persist in diverse environments such as aquatic ecosystems, maintaining a reservoir in surface waters and becoming a serious risk to public health and animal production systems. It is conceivable that the T6SS could mediate in part this persistence since it has been shown that *S.* Typhimurium requires the T6SS_SPI-6_ to survive intracellularly in environmental amoebas such as *Dictyostelium discoideum* (Riquelme et al., 2016). Interestingly, in Chile some serotypes such as *S.* Infantis, *S.* Newport and *S.* Typhimurium have been frequently isolated in surface waters during the last decade, imposing a significant threat to human and animal health since these serotypes usually carry an arsenal of antimicrobial resistance genes (Chen et al., 2024a,b). These Chilean isolates could be an untapped reservoir of new T6SS effector proteins. Importantly, *Salmonella* strains isolated from surface waters in Chile will shed light not only on the vast arsenal of T6SS effector repertoire but could also provide insight into geographic adaptation of *Salmonella*.

In this study, we performed bioinformatic and comparative genomic analyses of a dataset of 695 *S. enterica* genomes representing 44 serotypes isolated from different environmental sources in Chile, mostly surface waters. Our analysis revealed that most genomes only harbor the SPI-6 T6SS gene cluster, and that within its variable region 3 (VR3) we found four new candidate T6SS effectors with predicted nuclease activity. Noteworthy, many putative SPI-6 rearrangement hotspot (Rhs) effectors identified in this study harbor C-terminal extensions with unknown function. Overall, the diversity and distribution of T6SS effector proteins in Chilean *Salmonella* isolates suggest that different combinations of these proteins may contribute to the environmental fitness and pathogenic potential.

## Materials and methods

### Environmental samples and *Salmonella* isolation

Water samples were collected as part of a previous study (Toro et al., 2022) from sites in the Maipo, Mapocho, Claro and Lontué watersheds from the rivers themselves and connected tributaries, such as canals. Animal samples were collected as part of a previous study (Rivera et al., 2021) from industrial dairy farms, backyard systems and wild animals in the Región de Coquimbo, Región de Valparaíso, Región Metropolitana and Región del Libertador General Bernardo O’Higgins, Chile. A detailed description of sampling procedures, geographical location of samples and the procedure employed for *Salmonella* isolation from water an animal samples can be found elsewhere (Rivera et al., 2021; Toro et al., 2022).

### Whole genome sequencing, assembly, and quality control

For sequencing, each isolate was grown overnight at 37°C in tryptic soy broth and 1 mL of culture was used to purify DNA with the DNeasy Blood and Tissue Qiagen kit (Qiagen, CA, United States). Ratios of absorbance at 260 nm and 230 nm were obtained using a MaestroNano spectrophotometer (Maestro, Korea) and a QUBIT fluorimeter (Life Technologies, CA, United States). Libraries were prepared with the Illumina DNA Prep kit (Illumina, CA, United States) on the Sciclone G3 NGSx iQ Workstation (Perkin Elmer, MA, United States), and sequencing was performed on the Illumina NextSeq 2000 using the NextSeq 1000/2000 P2 reagents 300 cycles with the 150 paired-end chemistry (Illumina, CA, United States). Reads were examined for quality using FastQC (Galaxy version 0.69) (Wingett and Andrews, 2018) and trimmed using Trimmomatic (Galaxy version 0.36.4), with a minimum required quality of 20, averaging across 4 bases (Bolger et al., 2014). Processed reads were assembled using SPAdes (Galaxy version 3.11.1) with kmer sizes of 99 and 127, and careful correction (Bankevich et al., 2012). Assemblies were checked for quality using QUAST (Galaxy version 4.6.3) (Gurevich et al., 2013) and finally deposited in the NCBI Bioproject 560,080.^1^

*In silico* serotyping was carried out using SeqSero (Galaxy version 2.0.1) (Zhang et al., 2015) and SISTR (Galaxy version 1.0.2) (Yoshida et al., 2016). Finally, a single-nucleotide polymorphism (SNP) analysis was performed to identify clonality among isolates from the same sample. Clones were defined as isolates with genomes having 20 or fewer SNPs, as described by Pightling et al. (2018). According to this criterion, genome sequences from non-clonal isolates obtained from the same sample were selected for subsequent analysis. Thus, the genome sequence dataset analyzed in this study includes 695 *S. enterica* genomes from 44 distinct serotypes (Supplementary Table S1).

### Identification of T6SS gene clusters

The T6SS prediction tool from the Secret6 web server^2^ was used to identify T6SS gene clusters encoding the minimal 13 core components of a T6SS in each genome (Zhang et al., 2023). For selection of positive matches, a BLASTp 2.10.1+ identity threshold for T6SS prediction >30% and an E-value <0.0001 were used. These threshold values have been successfully used to identify T6SS gene clusters in *Salmonella* genomes (Amaya et al., 2022; Blondel et al., 2023).

### Identification of candidate T6SS effectors

To identify putative T6SS effectors encoded within the *Salmonella* genomes analyzed, each ORF encoded within the T6SS gene clusters identified was analyzed with the Bastion6 pipeline^3^ (Wang et al., 2018) excluding the 13 T6SS core components. ORFs presenting a Bastion6 score ≥ 0.7 were considered as candidate T6SS effectors. It is worth mentioning that a Bastion6 score ≥ 0.5 is routinely used as default setting for detection of T6SS effectors. However, we decided to use a score ≥ 0.7 to perform a more strict analysis. Each Bastion6 prediction was further analyzed using tools implemented in the Operon-Mapper web server^4^ (Taboada et al., 2018) to determine whether it was part of a single transcriptional unit that also encoded a putative immunity protein [i.e., a small protein with potential signal peptides (SignalP 6.0) and/or transmembrane domains (TMHMM 2.0)]. Conserved functional domains and motifs in the candidate T6SS effectors were identified using the PROSITE, NCBI-CDD, Motif-finder, and Pfam databases (Kanehisa et al., 2002; Sigrist et al., 2013; Finn et al., 2014; Lu et al., 2019) implemented in the GenomeNet search engine.^5^ An E-value cutoff score of 0.01 was used. In addition, for each putative effector and immunity protein identified, a biochemical functional prediction was performed by HMM homology searches using the HHpred HMM-HMM comparison tool^6^ (Zimmermann et al., 2017). Finally, a candidate T6SS effector was defined as “new” when it meets two criteria: (i) it includes at least one domain previously linked to antibacterial activity, and (ii) this domain has not been described as part of a T6SS effector in publicly available databases.

### Hierarchical clustering analysis of the new T6SS effectors

For hierarchical clustering analysis, a presence/absence matrix of each T6SS effector and candidate effector was constructed for each bacterial genome by means of BLASTn analyses and manual curation of the data (Supplementary Table S2). A 90% identity and 90% sequence coverage threshold was used to select positive matches, as done in previous analyses conducted by our group (Amaya et al., 2022; Blondel et al., 2023). The matrix generated was uploaded as a csv file to the online server MORPHEUS^7^ using default parameters (i.e., one minus Pearson’s correlation and average linkage method).

### Phylogenetic analyses of *Salmonella* T6SS gene clusters

TssC aminoacid sequences encoded in T6SS gene clusters from 605 *Salmonella* genomes were concatenated and aligned with ClustalW using the Molecular Evolutionary Genetics Analysis (MEGA) software version 7.0 (Kumar et al., 2016). A phylogenetic tree was built from the alignments obtained from MEGA by performing a bootstrap test of phylogeny (1,000 replications) using the maximum-likelihood method with a Jones-Taylor-Thornton correction model.

### Analysis of T6SS effectors distribution

The DNA sequence encoding each T6SS effector identified in this study was subjected to tBLASTx analyses to find orthologs in all *Salmonella* genome sequences deposited in the NCBI database (March, 2024) (Supplementary Tables S3, S4). For selection of positive matches, a 90% identity and 90% sequence coverage threshold was used. Conservation of sequences was determined by independent multiple sequence alignments using T-Coffee Expresso^8^ (Notredame et al., 2000), MAFFT^9^ (Katoh et al., 2017), and ESPript 3^10^ (Robert and Gouet, 2014). Comparative genomic analyses of T6SS gene clusters were performed using Mauve version 2.3.1^11^ (Darling et al., 2004) and EasyFig version 2.2.5^12^ (Sullivan et al., 2011). Nucleotide sequences were analyzed using Artemis version 18^13^ (Rutherford et al., 2000).

## Results

### T6SS gene clusters are widely distributed among Chilean *Salmonella* isolates

Previous analyses performed by our group have aimed in the identification of candidate T6SS effectors and cognate immunity proteins in *Salmonella* genomes deposited in public databases (Amaya et al., 2022; Blondel et al., 2023). In the present study, the analysis focused on genome sequences of *Salmonella* isolates obtained from different environmental sources in Chile, in order to shed light on the repertoire of T6SS candidate effectors present in *Salmonella* inhabiting our local geography. To this end, we analyzed a database of 695 high-quality sequenced *Salmonella* genomes from strains isolated from surface water and animal sources. Most isolates in this collection come from surface waters (674 isolates representing 34 serotypes), while 21 isolates representing only 8 serotypes were obtained from animal sources (14 in chicken, 3 in pigeon, 2 in pig and 2 in duck). Interestingly, the most frequently isolated serotypes were *S.* Infantis (*n* = 169), *S.* Agona (*n* = 71) and *S*. Newport (*n* = 11).

To identify T6SS gene clusters we used the T6SS prediction tool from the SecreT6 web server (see text footnote 2), which identified 622 putative T6SS gene clusters in 608 *Salmonella* genomes (Table 1; Supplementary Table S1). A more in-depth analysis revealed that these T6SS gene clusters correspond to those encoded in SPI-6, SPI-19 and SPI-21 (Table 1; Supplementary Figure S1). We could not identify T6SS gene clusters encoded in SPI-20 or SPI-22 in the genome of any isolate from our database. The SPI-6 T6SS gene cluster is widely distributed in 518 of the 695 genomes analyzed (74.5%), while the SPI-19 and SPI-21 T6SS gene clusters were only detected in 89 (12.8%) and 14 (2%) genomes, respectively (Table 1). Most isolates carried a unique T6SS gene cluster in SPI-6, SPI-19 or SPI-21, while a group of isolates belonging to serotype *S*. Livingstone harbors both SPI-6 and SPI-19 T6SS gene clusters. In contrast, no complete T6SS gene cluster was detected in isolates belonging to serotypes *S.* Enteritidis and *S*. Stanley.

**Table 1:** T6SS effectors and cognate immunity proteins encoded in T6SS gene clusters in Chilean *Salmonella* isolates.

Source of sample (Number of isolates)	T6SS gene cluster	T6SS effector^a^	Serotypes (Number of isolates with the corresponding T6SS effector)
Water (510), Chicken (5), Duck (2), Pig (2), Pigeon (3)	SPI-6	Tae2	*S*. Adelaide (4), *S*. Albany (1), *S*. Anatum (12), *S*. Bovismorbificans (37), *S*. Braenderup (4), *S*. Brandenburg (4), *S*. Cerro (12), *S.* Corvallis (9), *S*. Derby (1), *S*. Edinburgh (13), *S*. Give (4), *S*. Hadar (2), *S*. Heidelberg (1), *S.* Infantis (152), *S*. I -:b:1,5 (2), *S*. I 1,4,[5],12:d:- (1), *S*. I 1,4,[5],12:i:- (1), *S*. Johannesburg (1), *S*. Kentucky (1), *S*. Montevideo (2), *S*. Muenchen (5), *S*. Newport (1), *S*. Oranienburg (5), *S.* Panama (15), *S.* Paratyphi B (2), *S*. Sandiego (3), *S.* Santiago (4), *S*. Senftenberg (35), *S*. Soerenga (3), *S*. Tennessee (2), *S*. Thompson (10), *S.* Typhimurium (46), *S*. Worthington (4)
		Tae4	*S*. Adelaide (4), *S*. Albany (1), *S*. Anatum (12), *S*. Bovismorbificans (38), *S*. Braenderup (3), *S*. Cerro (11), *S.* Corvallis (10), *S*. Derby (1), *S*. Edinburgh (13), *S*. Give (4), *S*. Goldcoast (11), *S*. Hadar (2), *S*. Heidelberg (1), *S.* Infantis (151), *S*. I -:b:1,5 (3), *S*. I 1,4,[5],12:d:- (1), *S*. I 1,4,[5],12:i:- (1), *S*. Kentucky (1), *S*. Livingstone (23), *S*. Mbandaka (4), *S*. Montevideo (2), *S*. Muenchen (7), *S*. Newport (44), *S*. Oranienburg (5), *S.* Panama (15), *S.* Paratyphi B (2), *S*. Sandiego (3), *S.* Santiago (4), *S*. Senftenberg (33), *S*. Soerenga (3), *S*. Tennessee (2), *S*. Thompson (10), *S.* Typhimurium (46), *S*. Worthington (4)
		Tge2P	*S*. Adelaide (4), *S*. Bovismorbificans (38), *S*. Braenderup (3), *S.* Corvallis (10), *S*. Give (1), *S*. Hadar (2), *S*. Heidelberg (1), *S.* Infantis (152), *S*. I 1,4,[5],12:d:- (1), *S*. Johannesburg (1), *S*. Kentucky (1), *S*. Livingstone (7), *S*. Mbandaka (4), *S*. Muenchen (7), *S*. Newport (23), *S*. Sandiego (3), *S*. Senftenberg (35), *S*. Soerenga (3), *S*. Tennessee (2), *S*. Thompson (10), *S.* Typhimurium (1), *S*. Worthington (4)
		Tlde1	*S*. Adelaide (4), *S*. Albany (1), *S*. Anatum (12), *S*. Bovismorbificans (38), *S*. Braenderup (3), *S*. Brandenburg (4), *S*. Cerro (13), *S.* Corvallis (10), *S*. Derby (1), *S*. Goldcoast (11), *S*. Hadar (2), *S*. Heidelberg (1), *S.* Infantis (152), *S*. I 1,4,[5],12:d:- (1), *S*. I 1,4,[5],12:i:- (1), *S*. Johannesburg (1), *S*. Kentucky (1), *S*. Livingstone (25), *S*. Mbandaka (4), *S*. Muenchen (7), *S*. Newport (35), *S.* Paratyphi B (2), *S*. Sandiego (3), *S*. Senftenberg (1), *S*. Soerenga (3), *S*. Tennessee (2), *S*. Thompson (10), *S.* Typhimurium (46), *S*. Worthington (4)
		L-Ala, D-Glu endopeptidase	*S*. Bovismorbificans (37), *S*. Braenderup (1), *S*. Brandenburg (4), *S*. Edinburgh (13), *S*. Give (4), *S*. I -:b:1,5 (4), *S*. Johannesburg (1), *S*. Mbandaka (4), *S*. Montevideo (2), *S*. Newport (18), *S*. Oranienburg (5), *S.* Panama (15), *S*. Sandiego (3), *S*. Worthington (4)
		PgP	*S*. Braenderup (2)
		TseH-like	*S*. Edinburgh (13), *S*. I -:b:1,5 (6), *S.* Panama (15)
		Peptidase_M64	*S*. Braenderup (2), *S*. Give (4), *S*. Montevideo (2), *S*. Senftenberg (34), *S*. Tennessee (2)
		RhsA-HNHc	*S*. Tennessee (2)
		RhsA-Ntox47	*S*. Brandenburg (2), *S*. I 1,4,[5],12:i:- (1), *S.* Typhimurium (44)
		RhsA-Tox-HNH-EHHH	*S*. Braenderup (2), *S*. Derby (1)
		PAAR-RhsA-HNHc	*S*. Anatum (1), *S*. Edinburgh (1), *S*. Infantis (132), *S*. Kentucky (1), *S*. Senftenberg (1)
		PAAR-RhsA-Ntox47	*S*. Give (3), *S*. Livingstone (8), *S*. Muenchen (7), *S*. Newport (14), *S.* Panama (15), *S*. Sandiego (2)
		PAAR-RhsA-Tox-HNH-EHHH	*S*. Johannesburg (1), *S*. Tennessee (2)
		PAAR-RhsA-AHH	*S*. Goldcoast (11)
		PAAR-RhsA-GIY-YIG	*S*. Livingstone (8)
		RhsA-Tox-ART-HYD1	*S*. Thompson (7)
		PAAR-RhsA-Tox-ART-HYD1	*S*. Johannesburg (1)
		Rhs_main_	*S.* Typhimurium (36)
		**PAAR-RhsA-NucA_B**	*S*. Braenderup (1)
		**PAAR-RhsA-GH-E**	*S*. Albany (1)
		**PAAR-RhsA-CdiA**	*S*. Tennesse (2)
		**RhsA-CdiA**	*S*. Derby (1)
		**PAAR-RhsA-CT**	*S*. Adelaide (4), *S*. Braenderup (3), *S*. Brandenburg (2), *S*. Cerro (12), *S*. Derby (1), *S*. Edinburgh (5), *S*. Give (1), *S*. Hadar (2), *S*. Heidelberg (1), *S*. I 1,4,[5],12:i:- (1), *S*. Mbandaka (1), *S*. Montevideo (2), *S*. Newport (21), *S.* Paratyphi B (2), *S*. Sandiego (3), *S*. Soerenga (3), *S*. Thompson (10), *S.* Typhimurium (1), *S*. Worthington (4)
		**RhsA-CT**	*S*. Braenderup (2), *S*. Cerro (12), *S*. Edinburgh (5), *S*. Give (1), *S*. Hadar (2), *S*. Johannesburg (1), *S*. Thompson (9), *S.* Typhimurium (1)
Water (66)	SPI-19	**PAAR-RhsA-CT**	*S*. Agona (65), *S*. I 4:f,g,s:1,2 (1)
Water (13)	SPI-21	VgrG-PyocinS-HNHc	*S*. IIIb 35:i:z (1), *S*. IIIb 48:i:z (7)
		**Glucosaminidase**	*S*. IIIb 35:i:z (1), *S*. IIIb 48:i:z (11)
		**BTH_I2691**	*S*. IIIb 35:i:z (1)

a T6SS effectors and immunity proteins are designated according their formal name (in the case of those previously reported in the literature) or indicating the functional domains present in the predicted proteins (in the case of those having no formal names). New T6SS candidate effectors identified in this study are highlighted in **bold type**.

To identify high-confidence putative effectors encoded within every T6SS gene cluster detected, each ORF within these gene clusters was analyzed based on four criteria: (i) identification of candidate effectors through Bastion6 analysis (a bioinformatic tool that predicts T6SS effectors based on amino acid sequence, evolutionary information, and physicochemical properties); (ii) identification of putative immunity proteins by operon prediction (Operon-mapper; Taboada et al., 2018) and detection of signal peptides (SignalP 6.0) and transmembrane domains (TMHMM 2.0); (iii) identification of conserved functional domains associated with *bona fide* T6SS effectors (INTERPROSCAN, PROSITE, NCBI-CDD, MOTIF, and Pfam); and (iv) functional biochemical prediction using the HHpred HMM-HMM server. In addition, we further analyzed these T6SS gene clusters to identify potential unannotated ORFs that could encode putative effectors and cognate immunity proteins. Thus, our analysis revealed the presence of 6 new effector candidates encoded within the SPI-6 (4 effectors) and SPI-21 (2 effectors) T6SS gene clusters.

### The VR3 within the SPI-6 T6SS gene cluster of isolates from surface waters harbor four candidate T6SS effector proteins

Most T6SS effector proteins identified in *Salmonella* are encoded within three variable regions (VR1-3) of SPI-6 (Blondel et al., 2023). We have previously shown that the VR3 of SPI-6, located downstream of the *tssI* gene, exhibits the greatest diversity of *Salmonella* T6SS effectors (Blondel et al., 2023). This is mainly due to the presence of a variable number of Rhs effector proteins that harbor C-terminal extensions encoding endonuclease domains, such as DNases, RNases, and deaminases, as well as ADP-ribosyltransferases (Blondel et al., 2023).

**Figure 1:**
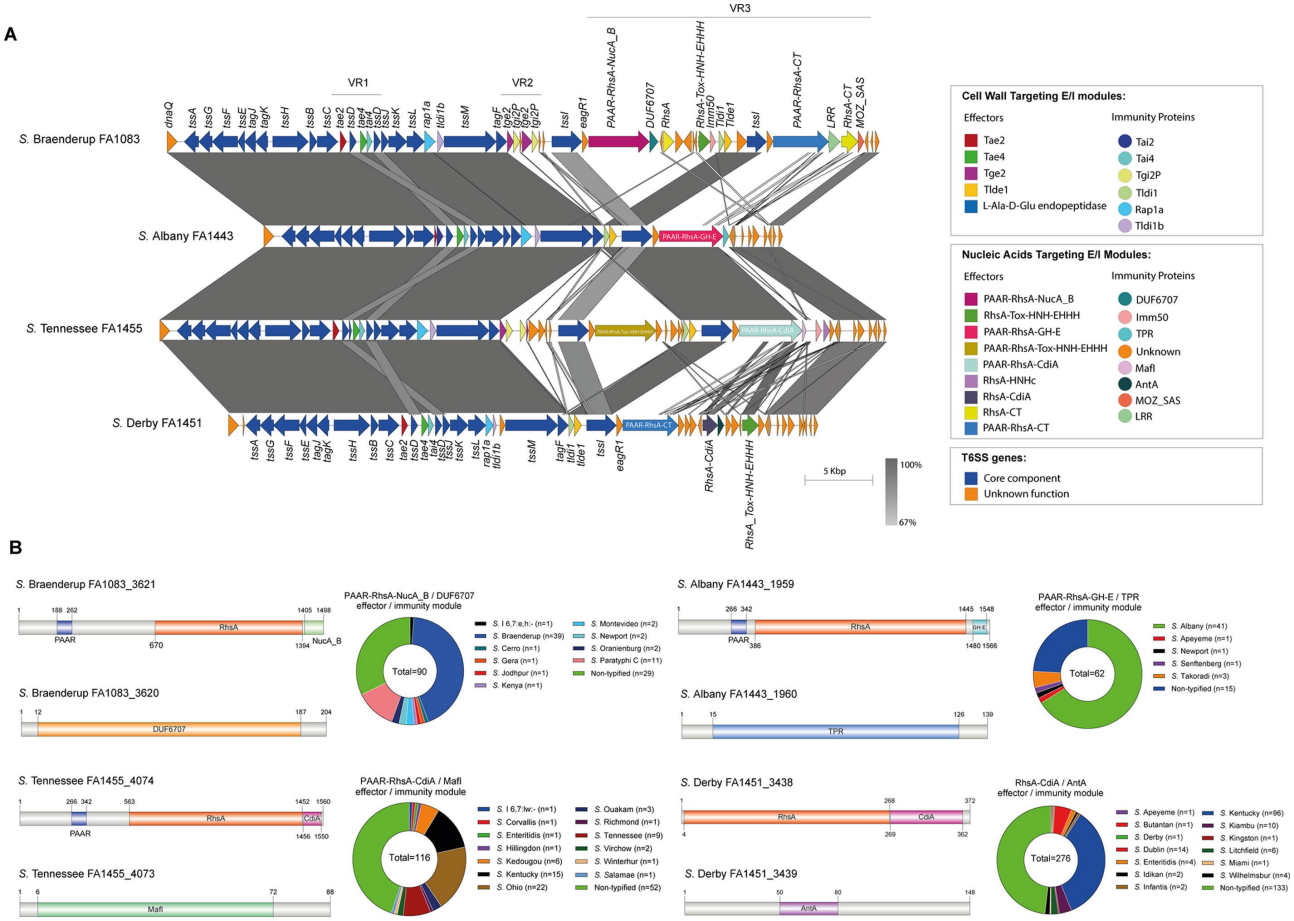
The SPI-6 T6SS gene cluster encodes new putative T6SS effector proteins. **(A)** Comparative genomic analysis of the SPI-6 T6SS cluster of *S.* Braenderup FA1083, *S.* Albany FA1443, *S*. Tennessee FA1455 and *S*. Derby FA1451. BLASTn sequence alignment was performed and visualized using EasyFig (Sullivan et al., 2011). **(B)** Schematic representation and distribution among *Salmonella* genomes of each new effector and immunity protein identified. ORFs encoding new E/I modules are highlighted in different colors according to the predicted functions. Homologs for each component were identified by BLASTn analyses as described in Materials and Methods.

Our analysis identified 4 new putative effector proteins and cognate immunity proteins (Table 2; Figure 1) encoded in the VR3 of SPI-6 distributed in isolates of serotypes *S*. Braenderup, *S*. Albany, *S*. Tennessee and *S*. Derby. Three of these candidates are specialized Rhs effector proteins with predicted nuclease activity, including 2 DNases and 1 RNase, while only one is a cargo Rhs effector with putative RNase activity (Table 2). The first putative effector (FA1083_3621 in *S.* Braenderup FA1083) is a large 1,498 amino acid Rhs protein that harbors an N-terminal PAAR domain and a C-terminal Nuclease A/Nuclease B (NucA_B) domain with predicted DNase activity (Table 2; Figure 1). It should be noted that *FA1083_3621* is predicted to be encoded in a bi-cistronic unit with *FA1083_3620* (Table 2). This latter ORF encodes a 204 amino acid protein with a DUF6707 domain that may correspond to the cognate immunity protein of FA1083_3621. The second candidate effector (FA1443_1959 in *S.* Albany FA1443) with predicted DNase activity also corresponds to a 1,566 amino acid Rhs protein that harbors an N-terminal PAAR domain and the putative GH-E domain in its C-terminal end (Table 2; Figure 1). The GH-E domain is found in members of the HNH/ENDO VII superfamily nuclease with conserved glycine, histidine and glutamate residues. This putative effector was also predicted to be co-transcribed with its respective putative immunity protein gene that encodes a tetratricopeptide repeat (TPR)-containing protein (FA1443_1960 in *S*. Albany FA1443). The third candidate effector (FA1455_4074 in *S*. Tennessee FA1455) is a 1,560 amino acid Rhs protein with a predicted N-terminal PAAR domain and a C-terminal contact-dependent growth inhibition protein A (CdiA) domain with putative RNase activity (Table 2; Figure 1). The gene encoding this candidate effector is predicted to be part of a bi-cistronic unit with *FA1455_4073*, encoding its putative immunity protein (Table 2; Figure 1). Of note, FA1455_4073 harbors a multiple adhesin family I (MafI) domain that is frequently found in cognate immunity proteins of bacterial toxin systems (Zhang et al., 2012). The fourth new candidate effector identified in this study is a 372 amino acid Rhs protein with a predicted CdiA domain in its C-terminal end (FA1451_3438 in *S*. Derby FA1451) (Table 2; Figure 1). *FA1451_3438* is predicted to be co-transcribed with *FA1451_3439*, encoding its cognate immunity protein (Table 2; Figure 1). FA1451_3439 harbors an anti-repressor A (AntA) domain usually found in phage anti-repressor proteins (Sandt et al., 2002). It is worth mentioning that the CdiA domain found in candidate effectors FA1455_4074 and FA1451_3438 has not been previously associated with any Rhs effector protein in *Salmonella*.

**Table 2:** New putative T6SS effectors and cognate immunity proteins encoded in the SPI-6 T6SS gene cluster of Chilean *Salmonella* isolates.

T6SS effector genes					Cognate T6SS immunity protein genes	
ORF(s)	Size (aa)	Serotype-isolate	Variable Region	Predicted activity/Domain	ORF(s)	TM or signal peptide/Domain^a^
Effectors targeting nucleic acids						
FA1083_3621	1,498	*S*. Braenderup FA1083	3	DNase/PAAR-RhsA-NucA_B	FA1083_3620	No/DUF6707
FA1443_1959	1,566	*S*. Albany FA1443	3	DNase/PAAR-RhsA-GH-E	FA1443_1960	No/TPR
FA1455_4074	1,560	*S*. Tennessee FA1455	3	RNase/PAAR-RhsA-CdiA	FA1455_4073	No/MafI
CFSAN035156_3316		*S*. Tennessee CFSAN035156			CFSAN035156_3317	
FA1451_3438	372	*S*. Derby FA1451	3	RNase/RhsA-CdiA	FA1451_3439	No/AntA

a Presence or absence of transmembrane domains (TM) or a signal peptide, and protein domains present in the putative immunity protein genes.

### The genetic structure and repertoire of effector proteins encoded in the SPI-6 T6SS gene cluster vary considerably among *Salmonella* isolates of the same serotype

It has been reported that the genetic structure of the T6SS gene clusters and the repertoire of effector proteins varies between different serotypes of *Salmonella* (Amaya et al., 2022; Blondel et al., 2023). Therefore we analyzed the genetic structure of SPI-6 and the distribution of previously identified effector proteins (Table 1; Supplementary Table S2). We identified 19 out of the 32 previously reported effectors encoded in the SPI-6 T6SS gene cluster. The three most frequently distributed T6SS effectors are encoded in VR1-2 of SPI-6. These effector proteins were Tae4 (34/36), Tae2 (32/36) and Tlde1 (29/36). In VR3, the region showing the greatest diversity of *Salmonella* T6SS effectors, the most prevalent effector proteins were PAAR-RhsA-Ntox47 (6/36) and PAAR-RhsA-HNHc (5/36).

**Figure 2:**
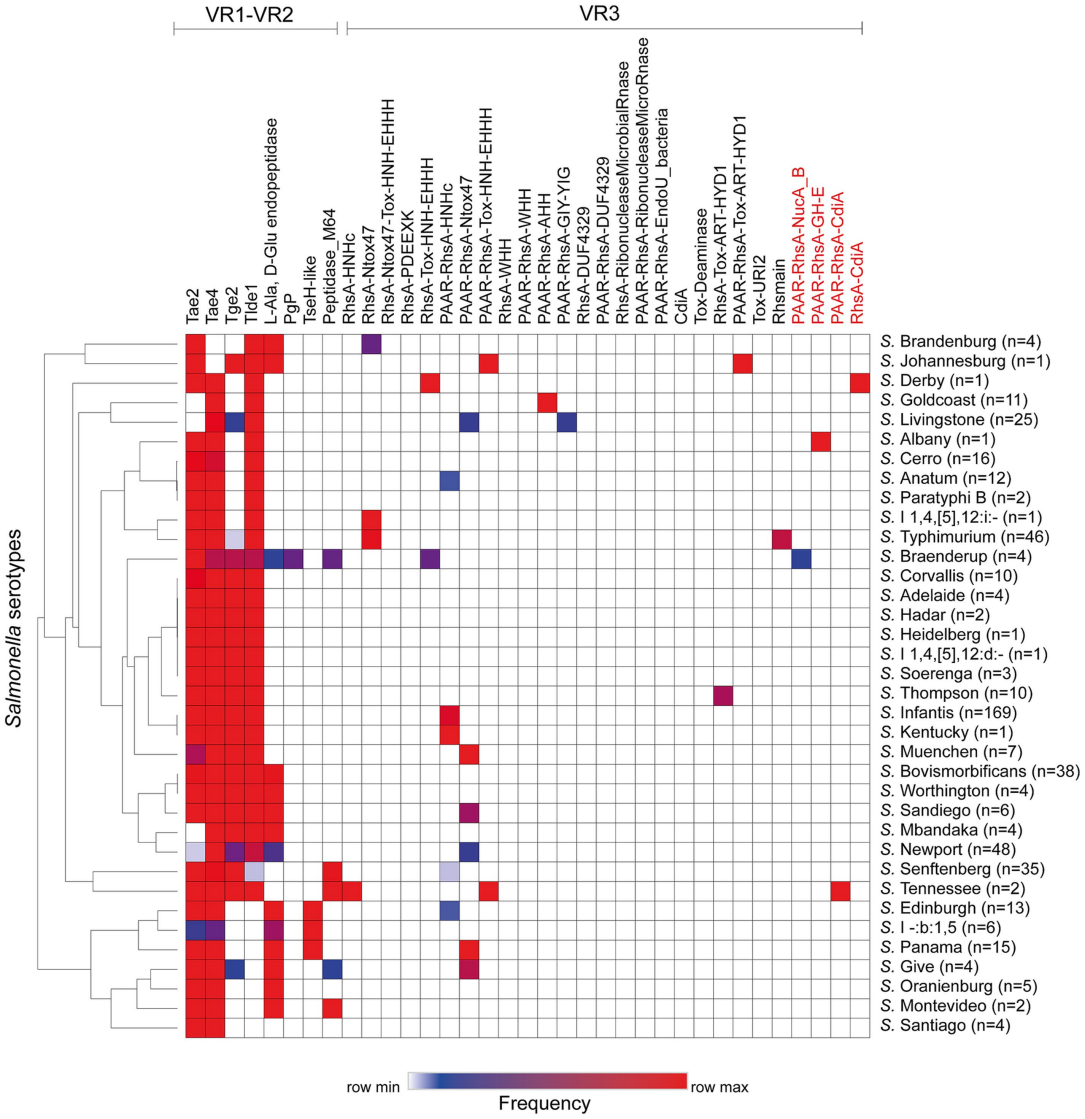
Prevalence of ORFs encoding T6SS effectors and candidate effectors in the SPI-6 T6SS gene cluster of Chilean *Salmonella* isolates. A hierarchical clustering analysis was conducted using MORPHEUS, as detailed in the Materials and Methods section. The color code in the heatmap indicates the frequency of a given ORF among all isolates of a particular *Salmonella* serotype. The names of new T6SS candidate effectors identified in this study are highlighted in red.

Next, we performed a hierarchical clustering analysis to shed lights into the distribution of effectors and candidate effectors encoded in the SPI-6 T6SS gene cluster identified (Supplementary Table S1). As illustrated in Figure 2, the four *bona fide* effectors encoded within VR1-2 (Tae2, Tae4, Tge2 and Tlde1) were the most conserved across the genomes of isolates representing 29 to 34 *Salmonella* serotypes. However, some of these effectors are missing from the genomes of all isolates from a few *Salmonella* serotypes. In VR3, the most prevalent effector protein was PAAR-RhsA-Ntox47, while PAAR-RhsA-AHH, PAAR-RhsA-GIY-YIG, PAAR-RhsA-Tox-ART-HYD1, RhsA-Tox-ART-HYD1 and RhsA-HNHc were the least prevalent. It is worth mentioning that a greater diversity of VR3-encoded effectors is observed in those serotypes that lack some of the more conserved VR1-2-encoded effectors (Figure 2).

**Figure 3:**
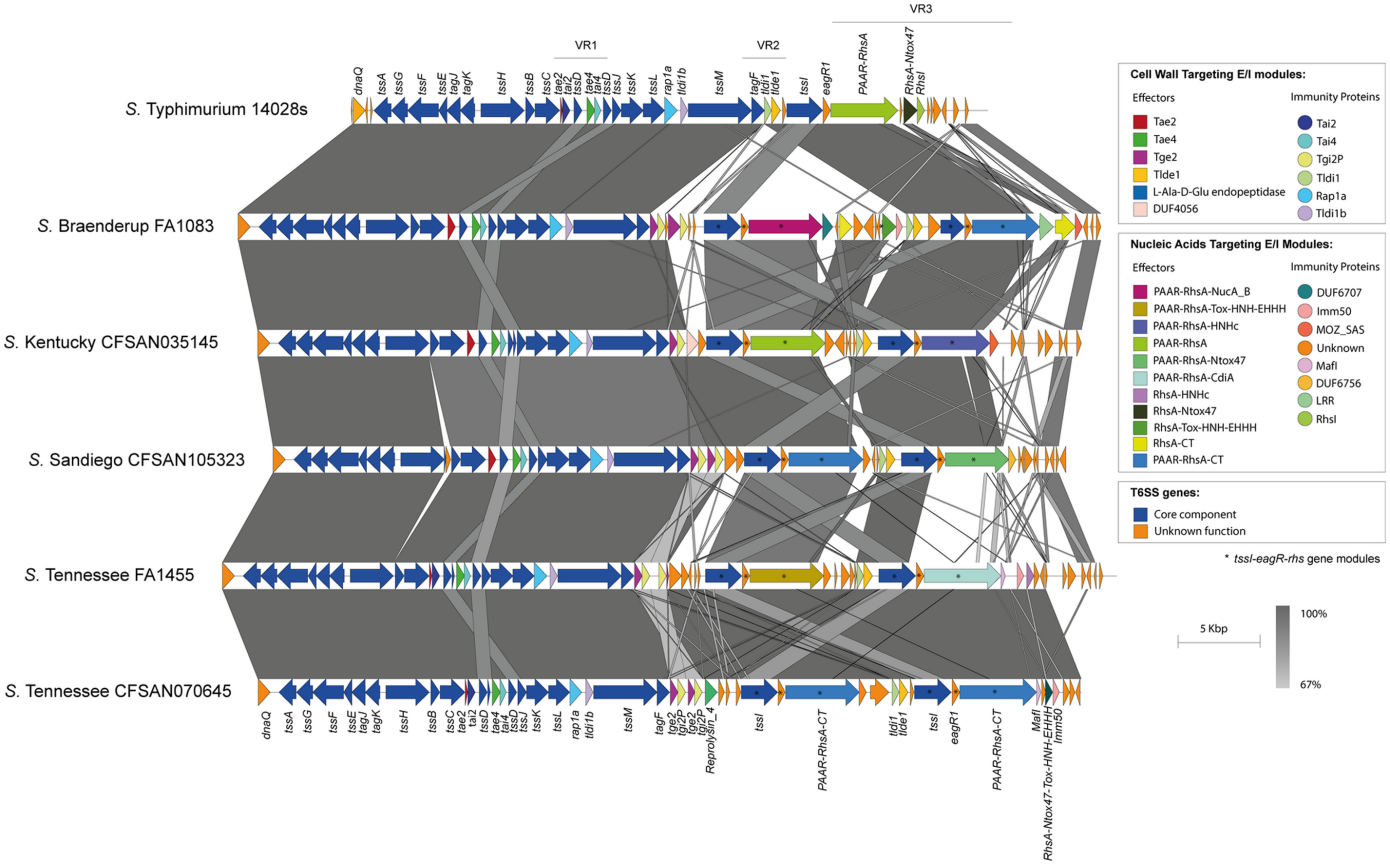
The SPI-6 T6SS gene cluster in a number of Chilean *Salmonella* isolates includes two *tssI*-*eagR*-*rhs* gene modules in VR3. Comparative genomic analysis of the SPI-6 T6SS cluster of *S.* Braenderup FA1083, *S*. Kentucky CFSAN035145, *S*. Sandiego CFSAN105323 and *S*. Tennessee FA1455 and CFSAN070645. BLASTn sequence alignment was performed and visualized using EasyFig (Sullivan et al., 2011). ORFs encoding E/I modules are highlighted in different colors according to the confirmed or predicted functions. The *tssI-eagR-rhs* gene modules of the SPI-6 T6SS gene cluster are demarked by asterisks. Grayscale represents the percentage of identity between nucleotide sequences. The SPI-6 T6SS gene cluster from *S.* Typhimurium 14028s was used for comparative purposes.

**Figure 4:**
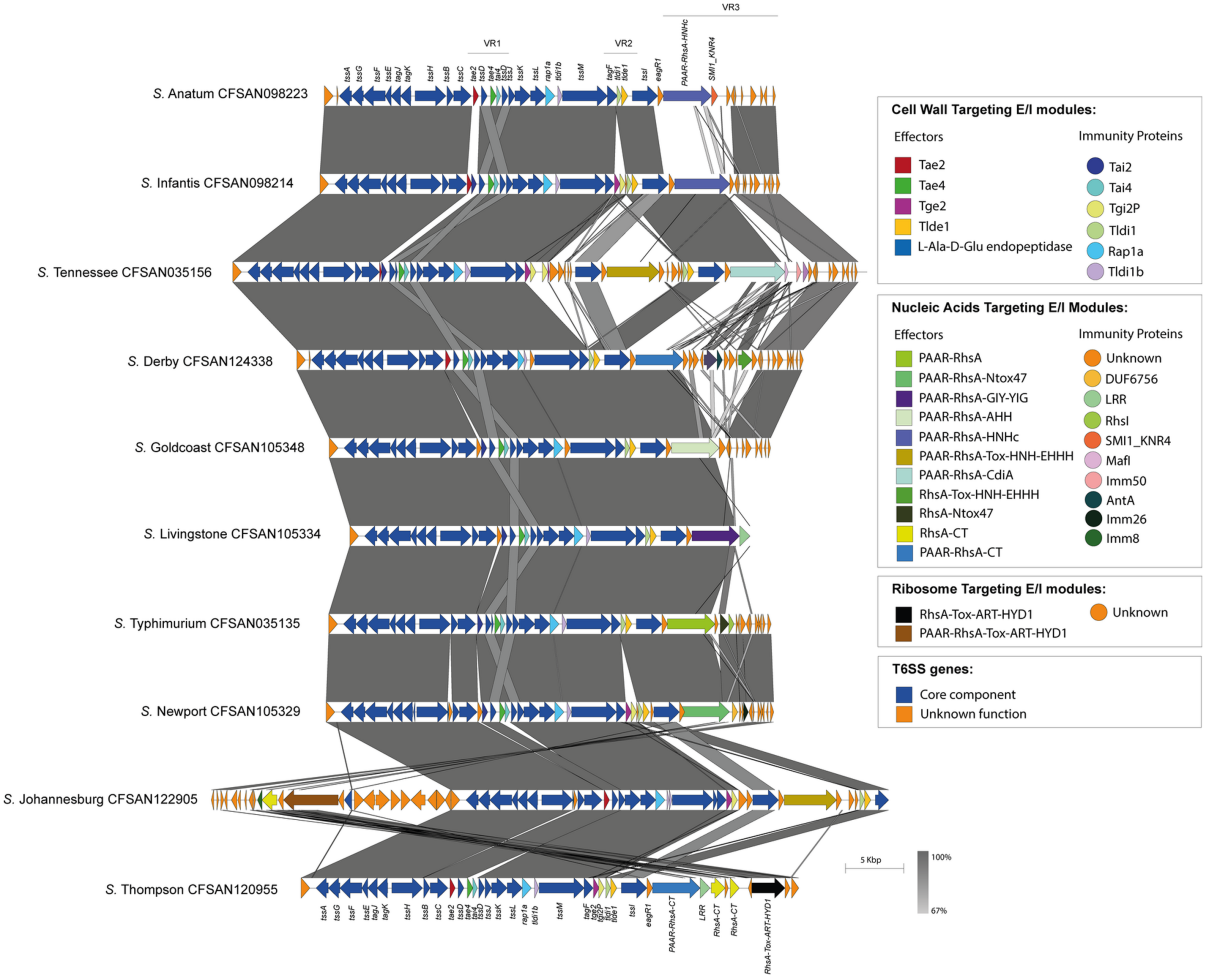
The T6SS_SPI-6_ effector repertoire varies among Chilean *Salmonella* isolates. Comparative genomic analysis of the SPI-6 T6SS cluster of selected *Salmonella* isolates representing different serotypes. BLASTn sequence alignment was performed and visualized using EasyFig (Sullivan et al., 2011). ORFs encoding T6SS core components are shown in blue. ORFs encoding E/I modules are highlighted in different colors according to the confirmed or predicted functions. Grayscale represents the percentage of identity between nucleotide sequences.

Analysis of genetic structure variation of the SPI-6 T6SS gene cluster between serotypes and between isolates of the same serotype revealed interesting observations. First, we identified a variable number of *tssI*-*eagR*-*rhs* gene modules encoded in VR3. A number of isolates from serotypes *S*. Braenderup, *S*. Kentucky, *S*. Sandiego and *S*. Tennessee harbor two *tssI*-*eagR*-*rhs* modules (Figure 3), while most isolates from serotypes carrying the SPI-6 T6SS gene cluster only harbor one *tssI*-*eagR*-*rhs* module (Figure 4). Remarkably, in *S*. Braenderup the genetic structure of SPI-6 differs between isolates CFSAN43223, FA0982 and FA1083. CFSAN43223 has only one *tssI*-*eagR*-*rhs* module, while FA0982 and FA1083 have two of these modules, as previously reported in *S*. Tennessee isolate CFSAN070645 (Blondel et al., 2023) (Figure 3; Supplementary Figure S2). Isolates FA0982 and FA1083 encode the RhsA-Tox-HNH-EHHH effector, as well as two other effectors harboring C-terminal ends with unknown function (PAAR-RhsA-CT and RhsA-CT). Additionally, isolate FA1083 encodes a new PAAR-RhsA-NucA_B effector with putative DNase activity, as described above (Figures 1, 3). It is important to note that isolate CFSAN43223 has an internal deletion within VR2 in comparison to isolates FA0982 and FA1083, and encodes only the Tlde1 effector. In contrast, isolates FA0982 and FA1083 encode two copies of the Tge2 effector in VR2 (Supplementary Figure S2). In *S*. Kentucky, our analysis of the single isolate present in the database (CFSAN035145) identified two *tssI*-*eagR*-*rhs* modules in VR3. These modules encode the PAAR-RhsA and PAAR-RhsA-HNHc effector proteins, respectively (Figure 3). Notably, the first *tssI-eagR-rhs* module has a high sequence identity with only one gene module previously reported in *S*. Tennessee CFSAN070645 (Blondel et al., 2023). Similarly, the second *tssI-eagR-rhs* module of *S*. Kentucky CFSAN035145 shows high sequence identity with the corresponding module encoded in VR3 of *S.* Typhimurium 14028s. Furthermore, *S*. Kentucky CFSAN035145 harbors an ORF with a predicted DUF4056 domain encoded in a bi-cistronic unit in VR2 never reported in *Salmonella*, which may constitute a new T6SS candidate effector (Figure 3). In *S*. Sandiego, the genetic structure of the SPI-6 T6SS gene cluster is conserved between isolates FA0894 and CFSAN105324, that harbor two *tssI-eagR-rhs* gene modules encoding a PAAR-RhsA-CT (C-terminal end with unknown function) and the PAAR-RhsA-Ntox47 effector proteins, respectively (Figure 3). A genomic comparative analysis of this latter effector with the corresponding T6SS effector in *S.* Typhimurium 14028s suggest that in isolates of serotype *S*. Sandiego the Rhs_main_ and RhsA-Ntox47 were at some point a single ORF that was later split due to the accumulation of nonsense mutations (Figure 3). Similar to *S*. Kentucky, the two *tssI-eagR-rhs* gene modules encoded in SPI-6 of *S*. Sandiego share high sequence identity with the corresponding gene modules encoded in *S*. Tennessee CFSAN070645 and *S.* Typhimurium 14028s, respectively (Figure 3). It is worth mentioning that Chilean *S*. Sandiego isolates harbor the Tae2 and Tae4 effector proteins encoded in VR1, as well as Tge2 and Tlde1 effectors encoded in VR2. Finally, in *S*. Tennessee, the genomic organization of the T6SS gene cluster encoded in SPI-6 is highly conserved not only among Chilean isolates but also among previously reported *S*. Tennessee isolates (Blondel et al., 2023) (Figure 3). Isolates of this serotype harbor two *tssI-eagR-rhs* gene modules encoding a PAAR-RhsA-Tox-HNH-EHHH and a PAAR-RhsA-CdiA T6SS effector proteins, respectively. Interestingly, unlike the other serotypes described above, these two *tssI-eagR-rhs* gene modules do not share any sequence identity with the corresponding module in *S.* Typhimurium 14028s. Altogether, these results suggest a distinct evolutionary origin of *tssI-eagR-rhs* gene modules within the SPI-6 T6SS gene cluster.

**Figure 5:**
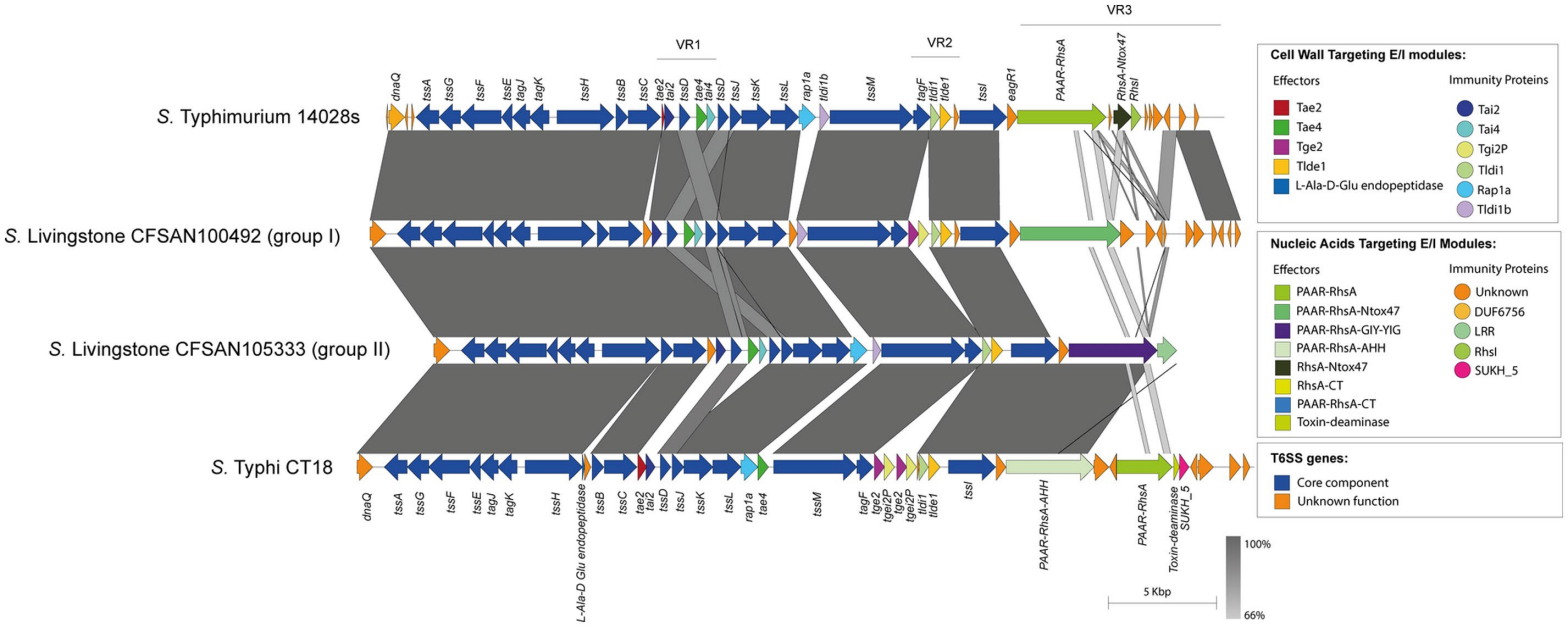
The genetic structure and repertoire of effector proteins encoded in the SPI-6 T6SS gene cluster vary among isolates of serotype *S*. Livingstone. Comparative genomic analysis of the SPI-6 T6SS cluster in *S*. Livingstone isolates. BLASTn sequence alignment was performed and visualized using EasyFig (Sullivan et al., 2011). ORFs encoding E/I modules are highlighted in different colors according to the confirmed or predicted functions. SPI-6 T6SS gene clusters from *S.* Typhimurium 14028s and *S.* Typhi CT18 were used for comparative purposes.

On the other hand, the isolates belonging to the remaining 32 serotypes only contain one *tssI-eagR-rhs* gene module encoded in the SPI-6 T6SS gene cluster. In these isolates, the distribution of known and new candidate effectors varies considerably, even among representatives of the same serotype. This is the case of *S*. Livingstone, where two groups of isolates are distinguished. In the first group, the VR3 encodes the PAAR-RhsA-Ntox47 effector, while isolates in the second group harbor the PAAR-RhsA-GIY-YIG effector (Figure 5). In addition, the VR2 in the first group encodes the Tge2 and Tlde1 effector proteins, while in the second group only encodes Tlde1 (Figure 5; Supplementary Table S1). Remarkably, the first group only harbor the SPI-6 T6SS gene cluster while the second group also encodes the SPI-19 T6SS gene cluster. Furthermore, the genetic structure of the SPI-6 T6SS cluster in the first group differs more with the T6SS gene cluster of *S.* Typhimurium 14028s when compared to the second group (Figure 5).

In isolates of serotype *S.* Give, the SPI-6 T6SS gene cluster shows structural differences in VR2 and VR3. In VR2, the isolate CFSAN043231 encodes the Tge2 and Peptidase M64 effector proteins, while other isolates (CFSAN119452, CFSAN119453, and CFSAN119454) carry a bi-cistronic unit encoding proteins with unknown function (Supplementary Figure S3). The putative immunity protein encoding-gene of this bi-cistronic unit harbors a DUF4229 domain found in integral membrane proteins (Wang et al., 2023). Another intriguing structural difference exists in VR3, where isolates CFSAN119452, CFSAN119453, and CFSAN119454 encode a PAAR-RhsA-Ntox47 effector protein, while isolate CFSAN043231 encodes a PAAR-RhsA-CT and an RhsA-CT, both harboring C-terminal ends with unknown functions (Supplementary Figure S3). Notably, the putative immunity protein encoding-gene of the RhsA-CT candidate effector harbors the Imm9 domain, which is frequently found in cognate immunity proteins of bacterial toxin systems with RNase activity (Zhang et al., 2012). Thus, the presence of the Imm9 domain in the putative immunity protein-encoding gene suggests that the C-terminal end of the RhsA-CT candidate effector has RNase activity.

The genetic organization of the SPI-6 T6SS gene cluster in *S*. Newport varies between two groups of isolates. In the first group, the isolates encode the PAAR-RhsA-Ntox47 effector in VR3 and the Tge2 effector in VR2. Furthermore, in VR3, these isolates also contain an ORF with a predicted DUF6769 domain encoded in a bi-cistronic unit with an ORF harboring an Imm26 domain, which is typically found in cognate immunity proteins of bacterial toxin systems with RNase activity (Zhang et al., 2012). The presence of the Imm26 domain in this ORF suggests that the DUF6769-containing protein is a candidate effector with RNase activity. On the other hand, isolates in the second group encode the PAAR-RhsA-CT effector in VR3 and do not encode the Tge2 effector in VR2 (Supplementary Figure S4). Of note, there is no sequence identity between the Rhs elements of both groups of isolates, suggesting a different origin. In addition, the sequence of the C-terminal end of the PAAR-RhsA-CT effector encoded in these isolates shows high sequence similarity with the Rhs element of *S.* Typhi CT18 (Supplementary Figure S4).

Similar findings were also identified in *S.* Edinburgh, where two groups of isolates were distinguished. In VR3, isolates in the first group encode the PAAR-RhsA-HNHc effector protein, while isolates in the second group encode the PAAR-RhsA-CT and RhsA-CT effectors with C-terminal ends with unknown function (Supplementary Figure S5). Notably, S. Edinburgh is one of the three serotypes in which the TseH-like effector is predicted to be encoded in VR2 (Supplementary Figure S5; Supplementary Table S1).

Finally, the SPI-6 T6SS gene cluster in the remaining 32 serotypes is highly conserved among isolates within the same serotype. However, the T6SS effector repertoire and its distribution varies considerably among these 32 serotypes (Figure 4). Notably, in VR3 these serotypes encode several T6SS effector proteins with different anti-bacterial activities, including putative DNases such as PAAR-RhsA-HNHc (*S.* Anatum, *S*. Edinburgh, *S.* Infantis, *S*. Kentucky, *S.* Senftenberg), RhsA-HNHc (*S*. Tennessee), RhsA-Tox-HNH-EHHH (*S*. Braenderup, *S.* Derby), PAAR-RhsA-Tox-HNH-EHHH (*S.* Johannesburg, *S*. Tennessee), PAAR-RhsA-AHH (*S.* Goldcoast) and PAAR-RhsA-GIY-YIG (*S*. Livingstone); putative RNases such as RhsA-Ntox47 (*S*. Brandenburg, *S*. I 1,4,[5],12:i:-, *S.* Typhimurium), PAAR-RhsA-Ntox47 (*S*. Give, *S*. Livingstone, *S.* Muenchen, *S*. Newport, *S.* Panama, *S*. Sandiego) and DUF4329 (*S*. Anatum); and putative ADP-ribosyltransferases such as PAAR-RhsA-Tox-ART-HYD1 (*S*. Johannesburg), RhsA-Tox-ART-HYD1 (*S.* Thompson) and RhsA_main_ (*S.* Typhimurium). Notably, 19 out of these 32 serotypes encode PAAR-RhsA-CT and RhsA-CT effectors harboring C-terminal ends with unknown function (Table 1; Figure 4). For instance, *S.* Johannesburg isolate CFSAN 122905 encodes an RhsA-CT candidate effector, along with a putative immunity protein harboring an Imm8 domain, which is commonly found in immunity proteins of bacterial toxin systems with RNase activity (Zhang et al., 2012). This result suggests that the C-terminal end of the RhsA-CT candidate effector has RNase activity.

### The SPI-19 Rhs effectors of Chilean *Salmonella* serotypes harbor C-terminal ends with protein domains of unknown function

The SPI-19 encodes a T6SS gene cluster present in some of the most prevalent *Salmonella* serotypes worldwide, such as *S*. Dublin*, S*. Agona, *S.* Weltevreden and *S.* Gallinarum, among others. Despite its contribution to intestinal colonization, antibacterial activity and cytotoxicity against macrophages (Blondel et al., 2013; Blondel et al., 2010; Pezoa et al., 2013, 2014; Schroll et al., 2019; Xian et al., 2020) no effector protein of this T6SS has been experimentally validated and tested. This is an important knowledge gap as infections triggered by these serotypes cause major economic problems in animal production and public health issues.

**Figure 6:**
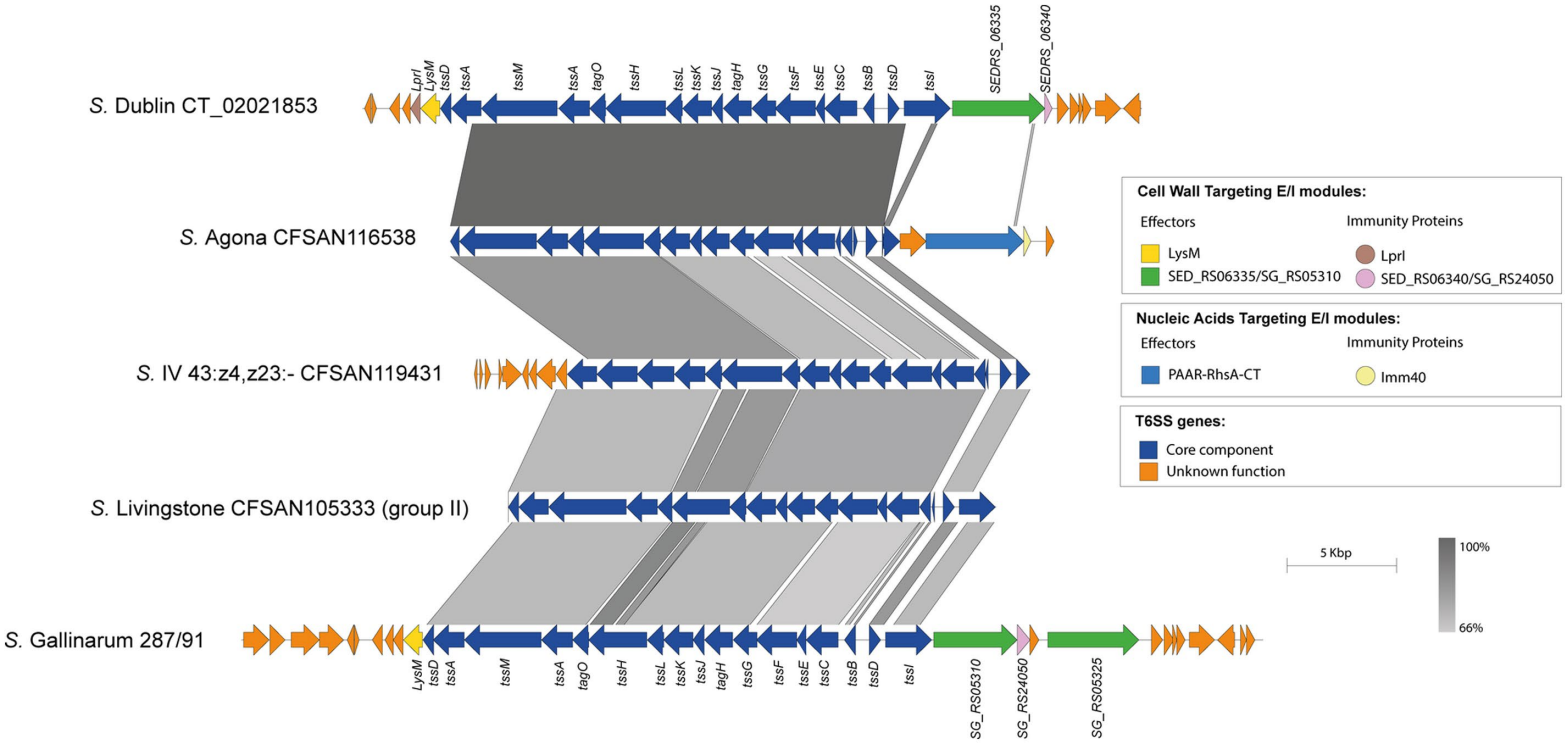
The SPI-19 T6SS gene cluster differs among Chilean *Salmonella* isolates and encodes putative T6SS Rhs effector proteins harboring C-terminal ends with domains of unknown function. Comparative genomic analysis of the SPI-19 T6SS gene cluster of *S.* Agona CFSAN116538, *S*. IV 43:z4,z23:- CFSAN119431 and *S*. Livingstone CFSAN105333. BLASTn sequence alignment was performed and visualized using EasyFig (Sullivan et al., 2011). ORFs encoding T6SS core components are shown in blue. ORFs encoding E/I modules are highlighted in different colors according to the confirmed or predicted functions. SPI-19 T6SS gene clusters from *S*. Dublin CT_02021853 (top) and *S.* Gallinarum 287/91 (bottom) were used for comparative purposes. Grayscale represents the percentage of identity between nucleotide sequences.

Our analysis identified the SPI-19 T6SS gene cluster in isolates representing 4 out of the 42 serotypes encoding T6SS. Of note, the genetic structure of this T6SS gene cluster differs among isolates of these 4 serotypes (Figure 6). In *S*. Agona, there are two groups of isolates that encode a PAAR-RhsA-CT effector and differ in the putative cognate immunity protein. The first group encodes a putative immunity protein with a predicted TPR domain, while in the second group this protein harbors an Imm40 domain that is frequently found in cognate immunity proteins of bacterial toxin systems with RNase activity (Zhang et al., 2012) (Figure 6). Therefore, the presence of the Imm40 domain in the putative immunity protein-encoding gene suggests that the C-terminal end of the PAAR-RhA-CT candidate effector has RNase activity. Of note, a single *S*. Agona isolate (CFSAN100497) lacks the SPI-19 T6SS gene cluster and harbors that encoded in SPI-6, which encodes the effector RhsA-Ntox47. This SPI-6 T6SS gene cluster exhibits high homology to the corresponding cluster in *S.* Typhimurium 14028s (Supplementary Figure S6).

In the case of the only isolate of serotype *S.* I 4:f,g,s:1,2 analyzed, the SPI-19 T6SS gene cluster exhibits high sequence conservation between the *tssK* and *tssI* core component genes with those encoded in the corresponding cluster of *S*. Dublin and *S.* Gallinarum (Figure 6). However, this serotype encodes a PAAR-RhsA-CT effector that has a different origin from the corresponding effector of *S*. Dublin and *S.* Gallinarum. Furthermore, the cognate immunity protein of this PAAR-RhsA-CT effector harbors an Imm40 domain (Zhang et al., 2012) (Figure 6), suggesting that the C-terminal end of PAAR-RhsA-CT has RNase activity.

Although we were not able to identify new effector candidates in the SPI-19 T6SS gene cluster of isolates belonging to serotypes *S*. IV 43:z4,z23:- and *S*. Livingstone, we found some features worth mentioning. In the case of serotype *S*. IV 43:z4,z23:-, the SPI-19 T6SS gene cluster is highly conserved among the 3 isolates analyzed. However, it shares lower degree of sequence identity with the corresponding gene cluster of *S*. Dublin and *S.* Gallinarum (Figure 6). The same was true for the group of 14 *S*. Livingstone isolates carrying both SPI-6 and SPI-19 T6SS gene clusters described above (Figure 6).

### The SPI-21 T6SS gene cluster from *S. enterica* subspecies *arizonae* and *diarizonae* encodes two candidate effectors

**Figure 7:**
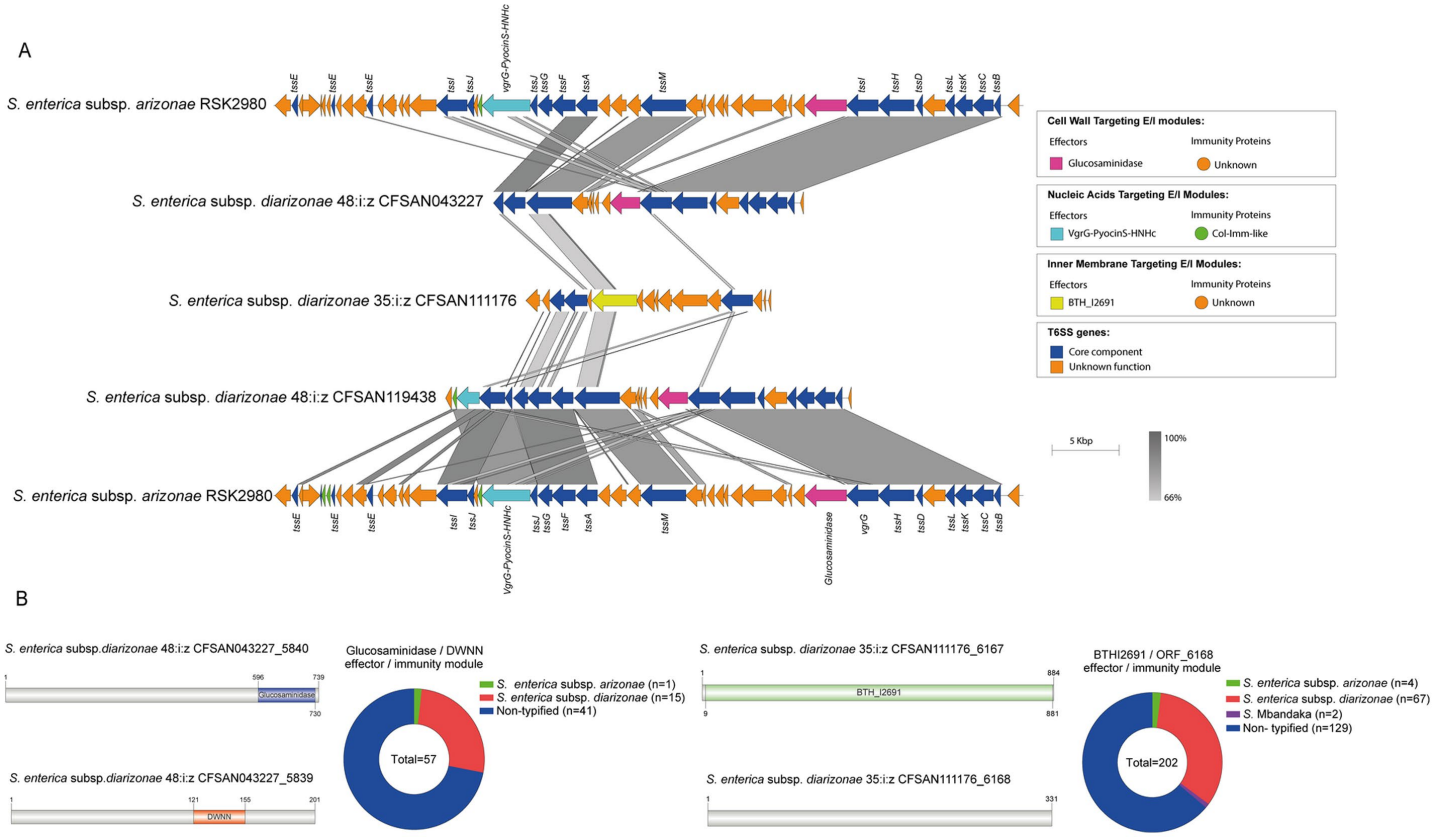
The SPI-21 T6SS gene cluster encodes new putative T6SS effector proteins. **(A)** Comparative genomic analysis of the SPI-21 T6SS cluster of *S. enterica* subsp. *diarizonae* 48:i:z CFSAN043227, *S. enterica* subsp. *diarizonae* 35:i:z CFSAN111176 and *S. enterica* subsp. *diarizonae* 48:i:z CFSAN119408. BLASTn sequence alignment was performed and visualized using EasyFig (Sullivan et al., 2011). SPI-21 T6SS gene cluster from *S. enterica* subsp. *arizonae* RSK2980 was used for comparative purposes. **(B)** Schematic representation and distribution among *Salmonella* genomes of each new effector and immunity protein identified. ORFs encoding new E/I modules are highlighted in different colors according to the predicted functions. Homologs for each component were identified by BLASTn analyses, as described in Materials and Methods.

To date there is very limited information regarding the effector proteins encoded in the SPI-21 T6SS gene cluster. Only one candidate effector has been described in *S. enterica* subsp. *arizonae* serotype 62:z4,z23:- reference strain RSK2980, which corresponds to a specialized VgrG protein with a C-terminal extension including a pyocin domain (S Type) (Blondel et al., 2009; Ho et al., 2017). Indeed, our bioinformatic analysis identified the VgrG-PyocinS-HNHc effector in most isolates of *S. enterica* subsp. *diarizonae* serotypes 48:i:z and 35:i:z (*S*. IIIb 48:i:z and *S*. IIIb 35:i:z, respectively) analyzed (Table 1; Figure 7A). The predicted cognate immunity protein of this candidate effector includes a inhibitory immunity protein of colicin DNase and pyocins (Col_Imm_like) domain, frequently present in immunity proteins of bacterial toxin systems (Zhang et al., 2012) (Figure 7A). Noteworthy, the SPI-21 T6SS gene cluster in all isolates of *S. enterica* subsp. *diarizonae* analyzed encodes a new candidate effector including a glucosaminidase domain with predicted peptidoglycan hydrolase activity (Table 3; Figure 7B). The predicted cognate immunity protein carries the domain with no name (DWNN). Furthermore, the SPI-21 T6SS gene cluster in the only isolate of *S*. IIIb 35:i:z analyzed (CFSAN111176) encodes a second new candidate effector with a predicted BTH_I2691 domain (Table 3; Figure 7B). Of note, BTH_I2691 is a T6SS effector protein originally described in *B. thailandensis* (Russell et al., 2012), which exhibits structural homology to colicin Ia (Parret et al., 2003). This suggests that the BTH_I2691 candidate effector protein may have membrane pore-forming activity. Finally, the SPI-21 T6SS gene cluster in all isolates of *S. enterica* subsp. *diarizonae* analyzed exhibit a relatively low degree of sequence identity with the corresponding gene cluster in *S. enterica* subsp. *arizonae* RSK2980 (Figure 7A).

**Table 3:** New putative T6SS effectors and cognate immunity proteins encoded in the SPI-21 T6SS gene cluster of Chilean *Salmonella* isolates.

T6SS effector genes				Cognate T6SS immunity protein genes	
ORF(s)	Size (aa)	Serotype-isolate	Predicted activity/Domain	ORF(s)	TM or signal peptide/Domain^a^
Effectors targeting peptidoglycan					
CFSAN043227_5840	739	*S*. IIIb 48:i:z CFSAN043227	Peptidoglycan hydrolase/Glucosaminidase	CFSAN043227_5839	No/DWNN
CFSAN119438_4687		*S*. IIIb 48:i:z CFSAN119438		CFSAN119438_4688	
Effectors targeting inner membrane					
CFSAN111176_6167	884	*S*. IIIb 35:i:z	Membrane-pore forming/BTH_I2691	CFSAN111176_6166	No/No

a Presence or absence of transmembrane domains (TM) or a signal peptide, and protein domains present in the putative immunity protein genes.

### Global genome-wide distribution analysis of the new candidate effectors identified in SPI-6 and SPI-21 T6SS gene clusters

The identification of 6 new candidate T6SS effectors, harboring protein domains frequently found in bacterial toxin systems, prompted us to determine their distribution across *Salmonella*. To this end, the nucleotide sequence corresponding to the ORF encoding each candidate effector was used in tBLASTx searches in publicly available *Salmonella* genome sequences deposited in the NCBI database (March, 2024) and the distribution of each effector was determined. Our analysis revealed that the new candidate effectors are distributed in a limited number of serotypes (Figures 1B, 7B). Indeed, effectors PAAR-RhsA-NucA_B, PAAR-RhsA-CdiA and RhsA-CdiA (encoded in the SPI-6 T6SS gene cluster) are distributed in 10 to 13 serotypes, while effector PAAR-RhsA-GH-E is distributed only in 5 serotypes (Figure 1B). In the case of the two candidate effectors encoded in the SPI-21 T6SS gene cluster, they are restricted to isolates of *S. enterica* subsp. *arizonae* and *S. enterica* subsp. *diarizonae* (Figure 7B).

## Discussion

The T6SS has emerged as a significant virulence and environmental fitness factor for Gram-negative bacteria. The T6SS is a versatile machine that delivers a wide range of effector proteins to bacterial and/or eukaryotic cells. As a result, it has become an essential weapon for mediating interbacterial competition and host-cell interactions for many bacterial pathogens. In *Salmonella*, five T6SS gene clusters have been identified within pathogenicity islands SPI-6, SPI-19, SPI-20, SPI-21, and SPI-22 (Blondel et al., 2009; Fookes et al., 2011) which belong to 4 different evolutionary lineages. However, information regarding the presence and distribution of T6SS gene clusters and their effector proteins is still limited, partly because most analyses have focused on a limited number of strains of a few serotypes.

In this study, to expand our knowledge regarding the distribution of T6SS gene clusters and the repertoire of T6SS effector proteins in *Salmonella*, we performed bioinformatic and comparative genomic analyses of a dataset including 695 *S. enterica* genomes, representing 44 serotypes isolated in Chile from different sources including surface waters, backyard systems and wildlife, among others. As expected, the SPI-6 T6SS gene cluster was the most prevalent in isolates of 36 different serotypes (87.48% of total *Salmonella* isolates), suggesting that the T6SS_SPI-6_ is one of the most critical molecular toolboxes for *Salmonella* pathogenicity and environmental fitness. Our analysis also confirmed previous observations suggesting that the T6SS_SPI-19_ is prevalent only in a subset of *Salmonella* serotypes, perhaps reflecting a contribution to *Salmonella* fitness in specialized environments and/or hosts (Blondel et al., 2009; Bao et al., 2019). Interestingly, we provide the first report on the presence of both SPI-6 and SPI-19 T6SS gene clusters in isolates of serotype *S*. Livingstone, as previously reported only in serotypes *S*. Dublin and *S*. Weltevreden (Blondel et al., 2009; Bao et al., 2019). Since the presence of multiple T6SSs in the same isolate is not common among *Salmonella* serotypes, it is still unclear how such multiplicity contributes to their environmental adaptation and/or pathogenic potential. Other T6SS gene clusters are restricted to specific serotypes. For instance, we identified the SPI-21 T6SS gene cluster only in isolates belonging to *S. enterica* subsp. *arizonae* and *S. enterica* subsp. *diarizonae,* as previously reported (Blondel et al., 2009; Bao et al., 2019). Regarding the repertoire of T6SS effector proteins of the Chilean *Salmonella* isolates, we identified 20 out of the 37 effectors previously identified in *Salmonella* (Blondel et al., 2009; Russell et al., 2012; Benz et al., 2013; Whitney et al., 2013; Koskiniemi et al., 2014; Sana et al., 2016; Ho et al., 2017; Sibinelli-Sousa et al., 2020; Amaya et al., 2022; Jurėnas et al., 2022; Lorente-Cobo et al., 2022; Hespanhol et al., 2022; Blondel et al., 2023). These effector proteins are distributed across 42 serotypes. It is notable that the content and distribution of T6SS effector proteins in local *Salmonella* isolates differs from previous reports (Blondel et al., 2023) and show differences between isolates of the same serotype. It is therefore tempting to speculate that diverse combinations of these proteins may have different effects on the environmental fitness, which could differentially contribute to geographic adaptations and/or pathogenic potential of *Salmonella* strains. Further experimental work is required to confirm this hypothesis.

One of these differences is exemplified by the variable number of *tssI-eagR-rhs* gene modules within the VR3 of the SPI-6 T6SS gene cluster. All these modules encode different T6SS effectors and candidate effectors. In *Salmonella*, 23 T6SS effector proteins with putative nuclease activity targeting DNA and RNA have been identified so far encoded in VR3 (Blondel et al., 2009; Koskiniemi et al., 2014; Ho et al., 2017; Amaya et al., 2022; Hespanhol et al., 2022; Blondel et al., 2023). In this work, we identified 4 new candidate effector proteins with potential nuclease activity within VR3 in SPI-6. This expands our knowledge regarding the versatility of the *Salmonella* T6SS effectors in targeting bacterial nucleic acids and highlights how they are one of the main bacterial targets of *Salmonella* T6SS effector proteins. Most of these effector proteins correspond to Rhs proteins with C-terminal ends including domains with predicted antibacterial activities, thus contributing to the diversification of the molecular targets of T6SSs in *Salmonella*. This was expected, given that previous studies have demonstrated that the VR3 of the SPI-6 T6SS gene cluster encodes a variable number of Rhs elements (Blondel et al., 2009; Amaya et al., 2022; Blondel et al., 2023) and that several Rhs proteins carry C-terminal polymorphic endonuclease domains, which are associated with T6SS effectors in *Salmonella* and other bacteria (Zhang et al., 2012; Koskiniemi et al., 2014; Amaya et al., 2022; Blondel et al., 2023).

Another exciting observation is that many of the putative SPI-6 and SPI-19 Rhs effectors identified in this study harbor C-terminal extensions with unknown function. However, the presence of putative immunity proteins encoded next to these Rhs proteins suggests that these effectors have an antibacterial activity. Thus, it is tempting to speculate that the arsenal of *Salmonella* T6SS effectors harbors a diverse array of protein domains with yet-to-be-discovered activities and bacterial targets.

Regarding the SPI-19 T6SS gene cluster, we could not identify new T6SS candidate effectors encoded in the genome of the local isolates analyzed. Of note, the previously identified T6SS candidate effectors, SED_RS06235 and SED_RS06335, encoded in the SPI-19 T6SS gene cluster of *S*. Dublin CT_02021853 harbor the LysM and metallopeptidase M91 domains, respectively (Amaya et al., 2022), both of which target the peptidoglycan layer.

The only known T6SS effector encoded in the SPI-21 T6SS gene cluster corresponds to VgrG-PyocinS-HNHc, which harbors putative nuclease activity and was previously identified in *S. enterica* subsp. *arizonae* 62:z4,z23:-s reference strain RSK2980 (Blondel et al., 2009; Ho et al., 2017). Noteworthy, the SPI-21 T6SS gene cluster from our local *Salmonella* isolates encodes two new candidate effector proteins. The first one includes a glucosaminidase domain with peptidoglycan hydrolase activity, while the second one harbors the BTH_I2691 domain with predicted membrane-pore forming activity. This is the first report of a T6SS candidate effector harboring the BTH_I2691 domain present in the *Salmonella* genus, which expands our knowledge on the molecules targeted by T6SS in competing bacteria. Furthermore, this BTH_I2691 domain exhibits predicted structural homology to colicin Ia, a bactericidal protein that forms a voltage-dependent channel in the inner membrane of target cells (Parret et al., 2003). These findings suggest that T6SS_SPI-21_ attacks different bacterial targets (i.e., nucleic acids, peptidoglycan and inner membrane), contributing to the fitness and virulence of both *S. enterica* subsp. *arizonae* and *S. enterica* subsp. *diarizonae*.

Finally, the distribution analysis of the six new T6SS candidate effectors identified in this study in *Salmonella* genomes from the NCBI database revealed that they are distributed in a limited number of serotypes, in contrast to the distribution previously reported for other T6SS candidate effectors in *Salmonella* (Blondel et al., 2023).

Altogether, our work broadens the repertoire of *Salmonella* T6SS effector proteins and provides evidence that the SPI-6, SPI-19 and SPI-21 T6SS gene clusters harbor a vast array of potential antibacterial effectors. This diversity is particularly evident in the VR3 of the SPI-6 T6SS gene cluster in our local *Salmonella* isolates, especially in those serotypes that lack some of the most conserved T6SS effectors encoded in VR2 (Figure 6). Finally, although this study increases the number of putative *Salmonella* antibacterial effectors against competing bacteria, it cannot be ruled out that those new candidate effectors targeting nucleic acids and cellular membranes may also affect eukaryotic cells. This represents a significant gap in our current understanding of the roles played by T6SS in host-pathogen interaction. In fact, no T6SS effector protein identified to date in *Salmonella* has been confirmed to target eukaryotic organisms, despite the clear contribution of *Salmonella* T6SSs to intracellular replication, survival and cytotoxicity inside the host immune cells (Mulder et al., 2012; Blondel et al., 2013; Schroll et al., 2019). Further research is required to address this issue.

## Data Availability

The datasets presented in this study can be found in online repositories. The names of the repository/repositories and accession number(s) can be found in the article/Supplementary material.

## Footnotes

^1^ https://www.ncbi.nlm.nih.gov/bioproject/560080

^2^ https://bioinfo-mml.sjtu.edu.cn/SecReT6/t6ss_prediction.php

^3^ https://bastion6.erc.monash.edu

^4^ https://biocomputo.ibt.unam.mx/operon_mapper

^5^ https://www.genome.jp

^6^ https://toolkit.tuebingen.mpg.de/tools/hhpred

^7^ https://software.broadinstitute.org/morpheus

^8^ https://tcoffee.crg.eu/apps/tcoffee/do:expresso

^9^ https://mafft.cbrc.jp/alignment/server/index.html

^10^ https://espript.ibcp.fr/ESPript/ESPript/

^11^ https://darlinglab.org/mauve/mauve.html

^12^ https://mjsull.github.io/Easyfig/files.html

^13^ https://sanger-pathogens.github.io/Artemis/Artemis/

## Supplementary material

SUPPLEMENTARY FIGURE S1 Phylogenetic analysis and E/I module composition of T6SS gene clusters in Chilean *Salmonella* isolates. Concatenated TssC aminoacid sequences encoded in the genome of 605 Chilean *Salmonella* isolates were aligned with ClustalW using MEGA version 7.0. Next, a maximum-likelihood phylogenetic tree was built from the alignment using a bootstrap test of phylogeny (1,000 replications) with a Jones-Taylor-Thornton correction model.

SUPPLEMENTARY FIGURE S2 The genetic structure and repertoire of effector proteins encoded in the SPI-6 T6SS gene cluster vary among isolates of serotype *S.* Braenderup. Comparative genomic analysis of the SPI-6 T6SS gene cluster in isolates of *S.* Braenderup. BLASTn sequence alignment was performed and visualized using EasyFig version 2.2.5 (Sullivan et al., 2011). ORFs encoding E/I modules are highlighted in different colors according to the confirmed or predicted functions. SPI-6 T6SS gene clusters from *S.* Typhimurium 14028s and *S.* Tennessee CFSAN070645 were used for comparative purposes.

SUPPLEMENTARY FIGURE S3 The genetic structure and repertoire of effector proteins encoded in the SPI-6 T6SS gene cluster vary among isolates of serotype *S.* Give. Comparative genomic analysis of the SPI-6 T6SS cluster in isolates of *S.* Give. BLASTn sequence alignment was performed and visualized using EasyFig version 2.2.5 (Sullivan et al., 2011). ORFs encoding E/I modules are highlighted in different colors according to the confirmed or predicted functions. SPI-6 T6SS gene clusters from *S.* Typhimurium 14028s and *S.* Typhi CT18 were used for comparative purposes.

SUPPLEMENTARY FIGURE S4 The genetic structure and repertoire of effector proteins encoded in the SPI-6 T6SS gene cluster vary among isolates of serotype *S.* Newport. Comparative genomic analysis of the SPI-6 T6SS cluster in isolates of *S.* Newport. BLASTn sequence alignment was performed and visualized using EasyFig version 2.2.5 (Sullivan et al., 2011). ORFs encoding E/I modules are highlighted in different colors according to the confirmed or predicted functions. SPI-6 T6SS gene cluster from *S.* Newport SL254 was used for comparative purposes.

SUPPLEMENTARY FIGURE S5 The genetic structure and repertoire of effector proteins encoded in the SPI-6 T6SS gene cluster vary among isolates of serotype *S.* Edinburgh. Comparative genomic analysis of the SPI-6 T6SS cluster in isolates of *S.* Edinburgh. BLASTn sequence alignment was performed and visualized using EasyFig version 2.2.5 (Sullivan et al., 2011). ORFs encoding E/I modules are highlighted in different colors according to the confirmed or predicted functions. SPI-6 T6SS gene clusters from *S.* Typhimurium 14028s and *S.* Typhi CT18 were used for comparative purposes.

SUPPLEMENTARY FIGURE S6 The SPI-6 T6SS gene cluster from *S.* Agona CFSAN100497 and *S.* Typhimurium 14028s share high sequence identity. Comparative genomic analysis of the SPI-6 T6SS gene cluster of S. Agona CFSAN100497 and *S.* Typhimurium 14028s. BLASTn sequence alignment was performed and visualized using EasyFig version 2.2.5 (Sullivan et al., 2011). ORFs encoding E/I modules are highlighted in different colors according to the confirmed or predicted functions.

SUPPLEMENTARY Table S1 Dataset of *Salmonella* genomes retrieved from Bioproject 560080 (https://www.ncbi.nlm.nih.gov/bioproject/560080).

SUPPLEMENTARY Table S2 Frequency of *Salmonella* isolates of a particular serotype harboring each effector and candidate effector encoded in SPI-6.

SUPPLEMENTARY Table S3 Distribution of SPI-6 T6SS effectors and candidate effectors in *Salmonella* genomes. The DNA sequence encoding each T6SS effector identified in this study was subjected to tBLASTx analyses to find orthologs in all *Salmonella* genome sequences deposited in the NCBI database (March 2024).

SUPPLEMENTARY Table S4 Distribution of SPI-21 T6SS effectors and candidate effectors in *Salmonella* genomes. The DNA sequence encoding each T6SS effector identified in this study was subjected to tBLASTx analyses to find orthologs in all *Salmonella* genome sequences deposited in the NCBI database (March 2024).

## References

[ref1] Ahmad S. Wang B. Walker M. D. Tran H.-K. R. Stogios P. J. Savchenko A.. (2019). An interbacterial toxin inhibits target cell growth by synthesizing (p)ppApp. Nature 575, 674–678. doi: 10.1038/s41586-019-1735-9, PMID: 31695193 PMC6883173

[ref2] Amaya F. A. Blondel C. J. Barros-Infante M. F. Rivera D. Moreno-Switt A. I. Santiviago C. A.. (2022). Identification of type VI secretion systems effector proteins that contribute to interbacterial competition in *Salmonella* Dublin. Front. Microbiol. 13:811932. doi: 10.3389/fmicb.2022.811932, PMID: 35222335 PMC8867033

[ref3] Bankevich A. Nurk S. Antipov D. Gurevich A. A. Dvorkin M. Kulikov A. S.. (2012). SPAdes: a new genome assembly algorithm and its applications to single-cell sequencing. J. Comput. Biol. 19, 455–477. doi: 10.1089/cmb.2012.0021, PMID: 22506599 PMC3342519

[ref4] Bao H. Zhao J.-H. Zhu S. Wang S. Zhang J. Wang X.-Y.. (2019). Genetic diversity and evolutionary features of type VI secretion systems in *Salmonella*. Future Microbiol. 14, 139–154. doi: 10.2217/fmb-2018-0260 30672329

[ref5] Benz J. Reinstein J. Meinhart A. (2013). Structural insights into the effector – immunity system Tae4/Tai4 from *Salmonella typhimurium*. PLoS One 8:e67362. doi: 10.1371/journal.pone.0067362, PMID: 23826277 PMC3695027

[ref6] Berni B. Soscia C. Djermoun S. Ize B. Bleves S. (2019). A type VI secretion system trans-kingdom effector is required for the delivery of a novel antibacterial toxin in *Pseudomonas aeruginosa*. Front. Microbiol. 10:1218. doi: 10.3389/fmicb.2019.01218, PMID: 31231326 PMC6560169

[ref7] Blondel C. J. Amaya F. A. Bustamante P. Santiviago C. A. Pezoa D. (2023). Identification and distribution of new candidate T6SS effectors encoded in *Salmonella* Pathogenicity Island 6. Front. Microbiol. 14:1252344. doi: 10.3389/fmicb.2023.1252344, PMID: 37664116 PMC10469887

[ref8] Blondel C. J. Jiménez J. C. Contreras I. Santiviago C. A. (2009). Comparative genomic analysis uncovers 3 novel loci encoding type six secretion systems differentially distributed in *Salmonella* serotypes. BMC Genomics 10:354. doi: 10.1186/1471-2164-10-354, PMID: 19653904 PMC2907695

[ref9] Blondel C. J. Jiménez J. C. Leiva L. E. Alvarez S. A. Pinto B. I. Contreras F.. (2013). The type VI secretion system encoded in *Salmonella* Pathogenicity Island 19 is required for *Salmonella enterica* serotype Gallinarum survival within infected macrophages. Infect. Immun. 81, 1207–1220. doi: 10.1128/iai.01165-12, PMID: 23357385 PMC3639620

[ref10] Blondel C. J. Yang H.-J. Castro B. Chiang S. Toro C. S. Zaldívar M.. (2010). Contribution of the type VI secretion system encoded in SPI-19 to chicken colonization by *Salmonella enterica* serotypes Gallinarum and Enteritidis. PLoS One 5:e11724. doi: 10.1371/journal.pone.0011724, PMID: 20661437 PMC2908676

[ref11] Bolger A. M. Lohse M. Usadel B. (2014). Trimmomatic: a flexible trimmer for Illumina sequence data. Bioinformatics 30, 2114–2120. doi: 10.1093/bioinformatics/btu170, PMID: 24695404 PMC4103590

[ref12] Brackmann M. Nazarov S. Wang J. Basler M. (2017). Using force to punch holes: mechanics of contractile nanomachines. Trends Cell Biol. 27, 623–632. doi: 10.1016/j.tcb.2017.05.003, PMID: 28602424

[ref13] Chassaing B. Cascales E. (2018). Antibacterial weapons: targeted destruction in the microbiota. Trends Microbiol. 26, 329–338. doi: 10.1016/j.tim.2018.01.006 29452951

[ref14] Chen Z. Moreno-Switt A. I. Reyes-Jara A. Delgado-Suarez E. Adell A. D. Oliveira C. J. B.. (2024a). A multicenter genomic epidemiological investigation in Brazil, Chile, and Mexico reveals the diversity and persistence of *Salmonella* populations in surface waters. mBio 15:e0077724. doi: 10.1128/mbio.00777-24, PMID: 38920393 PMC11253603

[ref15] Chen Z. Toro M. Moreno-Switt A. I. Adell A. D. Delgado-Suárez E. J. Bonelli R. R.. (2024b). Unveiling the genomic landscape of *Salmonella enterica* serotypes Typhimurium, Newport, and Infantis in Latin American surface waters: a comparative analysis. Microbiol. Spectr. 12:e0004724. doi: 10.1128/spectrum.00047-24, PMID: 38546218 PMC11064523

[ref16] Cherrak Y. Flaugnatti N. Durand E. Journet L. Cascales E. (2019). Structure and activity of the type VI secretion system. Microbiol. Spectr. 7:10-1128. doi: 10.1128/microbiolspec.PSIB-0031-2019, PMID: 31298206 PMC10957189

[ref17] Coulthurst S. (2019). The type VI secretion system: a versatile bacterial weapon. Microbiology 165, 503–515. doi: 10.1099/mic.0.000789, PMID: 30893029

[ref18] Coyne M. J. Comstock L. E. (2019). Type VI secretion systems and the gut microbiota. Microbiol. Spectr. 7:10-1128. doi: 10.1128/microbiolspec.PSIB-0009-2018, PMID: 30825301 PMC6404974

[ref19] Darling A. C. E. Mau B. Blattner F. R. Perna N. T. (2004). Mauve: multiple alignment of conserved genomic sequence with rearrangements. Genome Res. 14, 1394–1403. doi: 10.1101/gr.2289704, PMID: 15231754 PMC442156

[ref20] Diniz J. A. Coulthurst S. J. (2015). Intraspecies competition in *Serratia marcescens* is mediated by type VI-secreted Rhs effectors and a conserved effector-associated accessory protein. J. Bacteriol. 197, 2350–2360. doi: 10.1128/jb.00199-15, PMID: 25939831 PMC4524185

[ref21] Durand E. Cambillau C. Cascales E. Journet L. (2014). VgrG, Tae, Tle, and beyond: the versatile arsenal of type VI secretion effectors. Trends Microbiol. 22, 498–507. doi: 10.1016/j.tim.2014.06.004, PMID: 25042941

[ref22] Finn R. D. Bateman A. Clements J. Coggill P. Eberhardt R. Y. Eddy S. R.. (2014). Pfam: the protein families database. Nucleic Acids Res. 42, D222–D230. doi: 10.1093/nar/gkt1223, PMID: 24288371 PMC3965110

[ref23] Fookes M. Schroeder G. N. Langridge G. C. Blondel C. J. Mammina C. Connor T. R.. (2011). *Salmonella bongori* provides insights into the evolution of the salmonellae. PLoS Pathog. 7:e1002191. doi: 10.1371/journal.ppat.1002191, PMID: 21876672 PMC3158058

[ref24] Gurevich A. Saveliev V. Vyahhi N. Tesler G. (2013). QUAST: quality assessment tool for genome assemblies. Bioinformatics 29, 1072–1075. doi: 10.1093/bioinformatics/btt086, PMID: 23422339 PMC3624806

[ref25] Hespanhol J. T. Sanchez-Limache D. E. Nicastro G. G. Mead L. Llontop E. E. Chagas-Santos G.. (2022). Antibacterial T6SS effectors with a VRR-Nuc domain are structure-specific nucleases. eLife 11:e82437. doi: 10.7554/eLife.82437 36226828 PMC9635880

[ref26] Ho B. T. Fu Y. Dong T. G. Mekalanos J. J. (2017). *Vibrio cholerae* type 6 secretion system effector trafficking in target bacterial cells. Proc. Natl. Acad. Sci. USA 114, 9427–9432. doi: 10.1073/pnas.1711219114, PMID: 28808000 PMC5584461

[ref27] Issenhuth-Jeanjean S. Roggentin P. Mikoleit M. Guibourdenche M. De Pinna E. Nair S.. (2014). Supplement 2008–2010 (no. 48) to the white–Kauffmann–Le minor scheme. Res. Microbiol. 165, 526–530. doi: 10.1016/j.resmic.2014.07.004, PMID: 25049166

[ref28] Jiang F. Waterfield N. R. Yang J. Yang G. Jin Q. (2014). A *Pseudomonas aeruginosa* type VI secretion phospholipase D effector targets both prokaryotic and eukaryotic cells. Cell Host Microbe 15, 600–610. doi: 10.1016/j.chom.2014.04.010, PMID: 24832454

[ref29] Jurėnas D. Rey M. Byrne D. Chamot-Rooke J. Terradot L. Cascales E. (2022). *Salmonella* antibacterial Rhs polymorphic toxin inhibits translation through ADP-ribosylation of EF-Tu P-loop. Nucleic Acids Res. 50, 13114–13127. doi: 10.1093/nar/gkac1162, PMID: 36484105 PMC9825190

[ref30] Kanehisa M. Goto S. Kawashima S. Nakaya A. (2002). The KEGG databases at GenomeNet. Nucleic Acids Res. 30, 42–46. doi: 10.1093/nar/30.1.42, PMID: 11752249 PMC99091

[ref31] Katoh K. Rozewicki J. Yamada K. D. (2017). MAFFT online service: multiple sequence alignment, interactive sequence choice and visualization. Brief. Bioinform. 20, 1160–1166. doi: 10.1093/bib/bbx108 PMC6781576 28968734

[ref32] Koskiniemi S. Garza-Sánchez F. Sandegren L. Webb J. S. Braaten B. A. Poole S. J.. (2014). Selection of orphan Rhs toxin expression in evolved *Salmonella enterica* serovar Typhimurium. PLoS Genet. 10:e1004255. doi: 10.1371/journal.pgen.1004255, PMID: 24675981 PMC3967940

[ref33] Kumar S. Stecher G. Tamura K. (2016). MEGA7: molecular evolutionary genetics analysis version 7.0 for bigger datasets. Mol. Biol. Evol. 33, 1870–1874. doi: 10.1093/molbev/msw054, PMID: 27004904 PMC8210823

[ref34] Lorente-Cobo N. Sibinelli-Sousa S. Biboy J. Vollmer W. Bayer-Santos E. Prehna G. (2022). Molecular characterization of the type VI secretion system effector Tlde1a reveals a structurally altered LD-transpeptidase fold. J. Biol. Chem. 298:102556. doi: 10.1016/j.jbc.2022.102556, PMID: 36183829 PMC9638812

[ref35] Lu S. Wang J. Chitsaz F. Derbyshire M. K. Geer R. C. Gonzales N. R.. (2019). CDD/SPARCLE: the conserved domain database in 2020. Nucleic Acids Res. 48, D265–D268. doi: 10.1093/nar/gkz991, PMID: 31777944 PMC6943070

[ref36] Ma A. T. Mekalanos J. J. (2010). In vivo actin cross-linking induced by *Vibrio cholerae* type VI secretion system is associated with intestinal inflammation. Proc. Natl. Acad. Sci. 107, 4365–4370. doi: 10.1073/pnas.0915156107 20150509 PMC2840160

[ref37] Ma J. Sun M. Dong W. Pan Z. Lu C. Yao H. (2017). PAAR-Rhs proteins harbor various C-terminal toxins to diversify the antibacterial pathways of type VI secretion systems. Environ. Microbiol. 19, 345–360. doi: 10.1111/1462-2920.13621, PMID: 27871130

[ref38] Monjarás Feria J. Valvano M. A. (2020). An overview of anti-eukaryotic T6SS effectors. Front. Cell. Infect. Microbiol. 10:584751. doi: 10.3389/fcimb.2020.584751, PMID: 33194822 PMC7641602

[ref39] Mulder D. T. Cooper C. A. Coombes B. K. (2012). Type VI secretion system-associated gene clusters contribute to pathogenesis of *Salmonella enterica* serovar Typhimurium. Infect. Immun. 80, 1996–2007. doi: 10.1128/iai.06205-11, PMID: 22493086 PMC3370595

[ref40] Notredame C. Higgins D. G. Heringa J. (2000). T-coffee: a novel method for fast and accurate multiple sequence alignment. J. Mol. Biol. 302, 205–217. doi: 10.1006/jmbi.2000.4042, PMID: 10964570

[ref41] Parret A. H. Schoofs G. Proost P. De Mot R. (2003). Plant lectin-like bacteriocin from a rhizosphere-colonizing *Pseudomonas* isolate. J. Bacteriol. 185, 897–908. doi: 10.1128/JB.185.3.897-908.2003, PMID: 12533465 PMC142807

[ref42] Pezoa D. Blondel C. J. Silva C. A. Yang H.-J. Andrews-Polymenis H. Santiviago C. A.. (2014). Only one of the two type VI secretion systems encoded in the *Salmonella enterica* serotype Dublin genome is involved in colonization of the avian and murine hosts. Vet. Res. 45:2. doi: 10.1186/1297-9716-45-2, PMID: 24405577 PMC3899618

[ref43] Pezoa D. Yang H.-J. Blondel C. J. Santiviago C. A. Andrews-Polymenis H. L. Contreras I. (2013). The type VI secretion system encoded in SPI-6 plays a role in gastrointestinal colonization and systemic spread of *Salmonella enterica* serovar Typhimurium in the chicken. PLoS One 8:e63917. doi: 10.1371/journal.pone.0063917, PMID: 23691117 PMC3653874

[ref44] Pightling A. W. Pettengill J. B. Luo Y. Baugher J. D. Rand H. Strain E. (2018). Interpreting whole-genome sequence analyses of foodborne bacteria for regulatory applications and outbreak investigations. Front. Microbiol. 9:1482. doi: 10.3389/fmicb.2018.01482, PMID: 30042741 PMC6048267

[ref45] Pissaridou P. Allsopp L. P. Wettstadt S. Howard S. A. Mavridou D. A. I. Filloux A. (2018). The *Pseudomonas aeruginosa* T6SS-VgrG1b spike is topped by a PAAR protein eliciting DNA damage to bacterial competitors. Proc. Natl. Acad. Sci. 115, 12519–12524. doi: 10.1073/pnas.1814181115, PMID: 30455305 PMC6298103

[ref46] Riquelme S. Varas M. Valenzuela C. Velozo P. Chahin N. Aguilera P.. (2016). Relevant genes linked to virulence are required for *Salmonella* Typhimurium to survive intracellularly in the social amoeba *Dictyostelium discoideum*. Front. Microbiol. 7:1305. doi: 10.3389/fmicb.2016.01305, PMID: 27602025 PMC4993766

[ref47] Rivera D. Allel K. Dueñas F. Tardone R. Soza P. Hamilton-West C.. (2021). Screening the presence of non-typhoidal *Salmonella* in different animal systems and the assessment of antimicrobial resistance. Animals (Basel) 11:1532. doi: 10.3390/ani11061532, PMID: 34074040 PMC8225015

[ref48] Robert X. Gouet P. (2014). Deciphering key features in protein structures with the new ENDscript server. Nucleic Acids Res. 42, W320–W324. doi: 10.1093/nar/gku316, PMID: 24753421 PMC4086106

[ref49] Russell A. B. Singh P. Brittnacher M. Bui N. K. Hood R. D. Carl M. A.. (2012). A widespread bacterial type VI secretion effector superfamily identified using a heuristic approach. Cell Host Microbe 11, 538–549. doi: 10.1016/j.chom.2012.04.007, PMID: 22607806 PMC3358704

[ref50] Rutherford K. Parkhill J. Crook J. Horsnell T. Rice P. Rajandream M.-A.. (2000). Artemis: sequence visualization and annotation. Bioinformatics 16, 944–945. doi: 10.1093/bioinformatics/16.10.944, PMID: 11120685

[ref51] Sana T. G. Flaugnatti N. Lugo K. A. Lam L. H. Jacobson A. Baylot V.. (2016). *Salmonella* Typhimurium utilizes a T6SS-mediated antibacterial weapon to establish in the host gut. Proc. Natl. Acad. Sci. 113, E5044–E5051. doi: 10.1073/pnas.1608858113, PMID: 27503894 PMC5003274

[ref52] Sandt C. H. Hopper J. E. Hill C. W. (2002). Activation of prophage *eib* genes for immunoglobulin-binding proteins by genes from the IbrAB genetic island of *Escherichia coli* ECOR-9. J. Bacteriol. 184, 3640–3648. doi: 10.1128/JB.184.13.3640-3648.2002, PMID: 12057959 PMC135156

[ref53] Schroll C. Huang K. Ahmed S. Kristensen B. M. Pors S. E. Jelsbak L.. (2019). The SPI-19 encoded type-six secretion-systems (T6SS) of *Salmonella enterica* serovars Gallinarum and Dublin play different roles during infection. Vet. Microbiol. 230, 23–31. doi: 10.1016/j.vetmic.2019.01.006, PMID: 30827393

[ref54] Sibinelli-Sousa S. de Araújo-Silva A. L. Hespanhol J. T. Bayer-Santos E. (2022). Revisiting the steps of *Salmonella* gut infection with a focus on antagonistic interbacterial interactions. FEBS J. 289, 4192–4211. doi: 10.1111/febs.16211, PMID: 34546626

[ref55] Sibinelli-Sousa S. Hespanhol J. T. Nicastro G. G. Matsuyama B. Y. Mesnage S. Patel A.. (2020). A family of T6SS antibacterial effectors related to l,d-transpeptidases targets the peptidoglycan. Cell Rep. 31:107813. doi: 10.1016/j.celrep.2020.107813 32579939

[ref56] Sigrist C. J. A. de Castro E. Cerutti L. Cuche B. A. Hulo N. Bridge A.. (2013). New and continuing developments at PROSITE. Nucleic Acids Res. 41, D344–D347. doi: 10.1093/nar/gks1067, PMID: 23161676 PMC3531220

[ref57] Silverman J. M. Agnello D. M. Zheng H. Andrews B. T. Li M. Catalano C. E.. (2013). Haemolysin coregulated protein is an exported receptor and chaperone of type VI secretion substrates. Mol. Cell 51, 584–593. doi: 10.1016/j.molcel.2013.07.025, PMID: 23954347 PMC3844553

[ref58] Srikannathasan V. English G. Bui N. K. Trunk K. O’Rourke P. E. F. Rao V. A.. (2013). Structural basis for type VI secreted peptidoglycan dl-endopeptidase function, specificity and neutralization in *Serratia marcescens*. Acta Crystallogr. D Biol. Crystallogr. 69, 2468–2482. doi: 10.1107/s0907444913022725, PMID: 24311588 PMC3852654

[ref59] Sullivan M. J. Petty N. K. Beatson S. A. (2011). Easyfig: a genome comparison visualizer. Bioinformatics 27, 1009–1010. doi: 10.1093/bioinformatics/btr039, PMID: 21278367 PMC3065679

[ref60] Taboada B. Estrada K. Ciria R. Merino E. (2018). Operon-mapper: a web server for precise operon identification in bacterial and archaeal genomes. Bioinformatics 34, 4118–4120. doi: 10.1093/bioinformatics/bty496, PMID: 29931111 PMC6247939

[ref61] Tang J. Y. Bullen N. P. Ahmad S. Whitney J. C. (2018). Diverse NADase effector families mediate interbacterial antagonism via the type VI secretion system. J. Biol. Chem. 293, 1504–1514. doi: 10.1074/jbc.ra117.000178, PMID: 29237732 PMC5798281

[ref62] Ting S.-Y. Bosch D. E. Mangiameli S. M. Radey M. C. Huang S. Park Y.-J.. (2018). Bifunctional immunity proteins protect bacteria against FtsZ-targeting ADP-ribosylating toxins. Cell 175, 1380–1392.e14. doi: 10.1016/j.cell.2018.09.037, PMID: 30343895 PMC6239978

[ref63] Toro M. Weller D. Ramos R. Diaz L. Alvarez F. P. Reyes-Jara A.. (2022). Environmental and anthropogenic factors associated with the likelihood of detecting *Salmonella* in agricultural watersheds. Environ. Pollut. 306:119298. doi: 10.1016/j.envpol.2022.119298, PMID: 35430308

[ref65] Uzzau S. Brown D. J. Wallis T. Rubino S. Leori G. Bernard S.. (2000). Host adapted serotypes of *Salmonella enterica*. Epidemiol. Infect. 125, 229–255. doi: 10.1017/s0950268899004379, PMID: 11117946 PMC2869595

[ref66] Wang J. Chitsaz F. Derbyshire M. K. Gonzales N. R. Gwadz M. Lu S.. (2023). The conserved domain database in 2023. Nucleic Acids Res. 51, D384–D388. doi: 10.1093/nar/gkac1096, PMID: 36477806 PMC9825596

[ref67] Wang J. Yang B. Leier A. Marquez-Lago T. T. Hayashida M. Rocker A.. (2018). Bastion6: a bioinformatics approach for accurate prediction of type VI secreted effectors. Bioinformatics 34, 2546–2555. doi: 10.1093/bioinformatics/bty155, PMID: 29547915 PMC6061801

[ref68] Whitney J. C. Beck C. M. Goo Y. A. Russell A. B. Harding B. N. Leon J. A. D.. (2014). Genetically distinct pathways guide effector export through the type VI secretion system. Mol. Microbiol. 92, 529–542. doi: 10.1111/mmi.12571, PMID: 24589350 PMC4049467

[ref69] Whitney J. C. Chou S. Russell A. B. Biboy J. Gardiner T. E. Ferrin M. A.. (2013). Identification, structure, and function of a novel type VI secretion peptidoglycan glycoside hydrolase effector-immunity pair. J. Biol. Chem. 288, 26616–26624. doi: 10.1074/jbc.m113.488320, PMID: 23878199 PMC3772208

[ref70] Whitney J. C. Quentin D. Sawai S. LeRoux M. Harding B. N. Ledvina H. E.. (2015). An interbacterial NAD(P)^+^ glycohydrolase toxin requires elongation factor Tu for delivery to target cells. Cell 163, 607–619. doi: 10.1016/j.cell.2015.09.027, PMID: 26456113 PMC4624332

[ref71] Wingett S. W. Andrews S. (2018). FastQ screen: a tool for multi-genome mapping and quality control. F1000Res 7:1338. doi: 10.12688/f1000research.15931.2, PMID: 30254741 PMC6124377

[ref72] Wood T. E. Howard S. A. Förster A. Nolan L. M. Manoli E. Bullen N. P.. (2019). The *Pseudomonas aeruginosa* T6SS delivers a periplasmic toxin that disrupts bacterial cell morphology. Cell Rep. 29, 187–201.e7. doi: 10.1016/j.celrep.2019.08.094, PMID: 31577948 PMC6899460

[ref73] Xian H. Yuan Y. Yin C. Wang Z. Ji R. Chu C.. (2020). The SPI-19 encoded T6SS is required for *Salmonella* Pullorum survival within avian macrophages and initial colonization in chicken dependent on inhibition of host immune response. Vet. Microbiol. 250:108867. doi: 10.1016/j.vetmic.2020.108867, PMID: 33010573

[ref74] Yoshida C. E. Kruczkiewicz P. Laing C. R. Lingohr E. J. Gannon V. P. Nash J. H.. (2016). The *Salmonella* in silico typing resource (SISTR): an open web-accessible tool for rapidly typing and subtyping draft *Salmonella* genome assemblies. PLoS One 11:e0147101. doi: 10.1371/journal.pone.0147101, PMID: 26800248 PMC4723315

[ref75] Zhang D. de Souza R. F. Anantharaman V. Iyer L. M. Aravind L. (2012). Polymorphic toxin systems: comprehensive characterization of trafficking modes, processing, mechanisms of action, immunity and ecology using comparative genomics. Biol. Direct 7:18. doi: 10.1186/1745-6150-7-18, PMID: 22731697 PMC3482391

[ref76] Zhang J. Guan J. Wang M. Li G. Djordjevic M. Tai C.. (2023). SecReT6 update: a comprehensive resource of bacterial type VI secretion systems. Sci. China Life Sci. 66, 626–634. doi: 10.1007/s11427-022-2172-x, PMID: 36346548

[ref77] Zhang S. Yin Y. Jones M. B. Zhang Z. Deatherage Kaiser B. L. Dinsmore B. A.. (2015). *Salmonella* serotype determination utilizing high-throughput genome sequencing data. J. Clin. Microbiol. 53, 1685–1692. doi: 10.1128/JCM.00323-15, PMID: 25762776 PMC4400759

[ref78] Zimmermann L. Stephens A. Nam S.-Z. Rau D. Kübler J. Lozajic M.. (2017). A completely reimplemented MPI bioinformatics toolkit with a new HHpred server at its core. J. Mol. Biol. 430, 2237–2243. doi: 10.1016/j.jmb.2017.12.007 29258817

