# Supplementary figures and images for: Genomic analysis of Salmonella isolated from surface water and animal sources in Chile reveals new T6SS effector protein candidates
Source: Front Microbiol. 2024 Dec 11;15:1496223. doi: 10.3389/fmicb.2024.1496223 (PMC11669294; doi:10.3389/fmicb.2024.1496223)

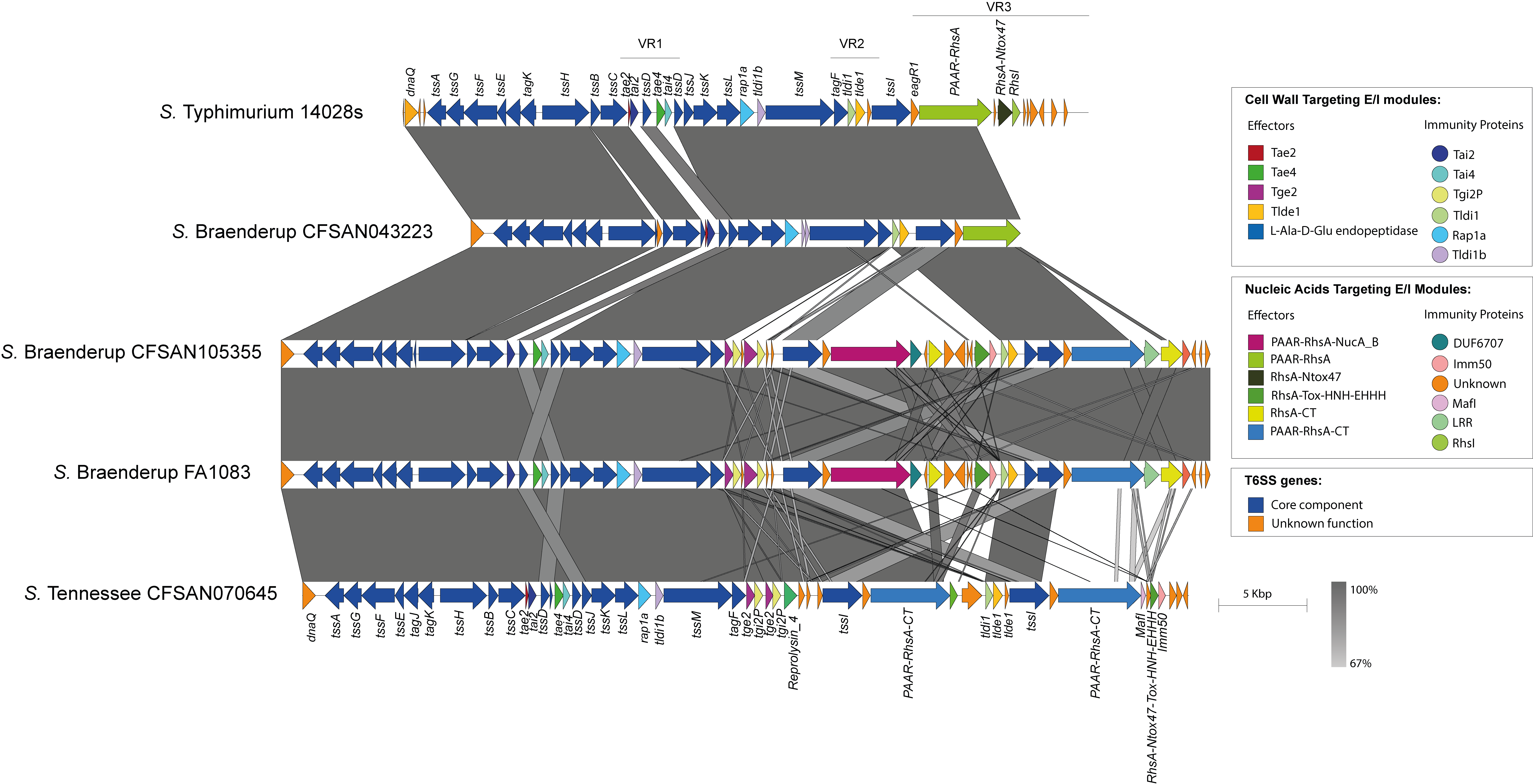

Supplement: SUPPLEMENTARY FIGURE S2 — The genetic structure and repertoire of effector proteins encoded in the SPI-6 T6SS gene cluster vary among isolates of serotype S. Braenderup. Comparative genomic analysis of the SPI-6 T6SS gene cluster in isolates of S. Braenderup. BLASTn sequence alignment was performed and visualized using EasyFig version 2.2.5 (Sullivan et al., 2011). ORFs encoding E/I modules are highlighted in different colors according to the confirmed or predicted functions. SPI-6 T6SS gene clusters from S. Typhimurium 14028s and S. Tennessee CFSAN070645 were used for comparative purposes. [file Image_2.TIF]

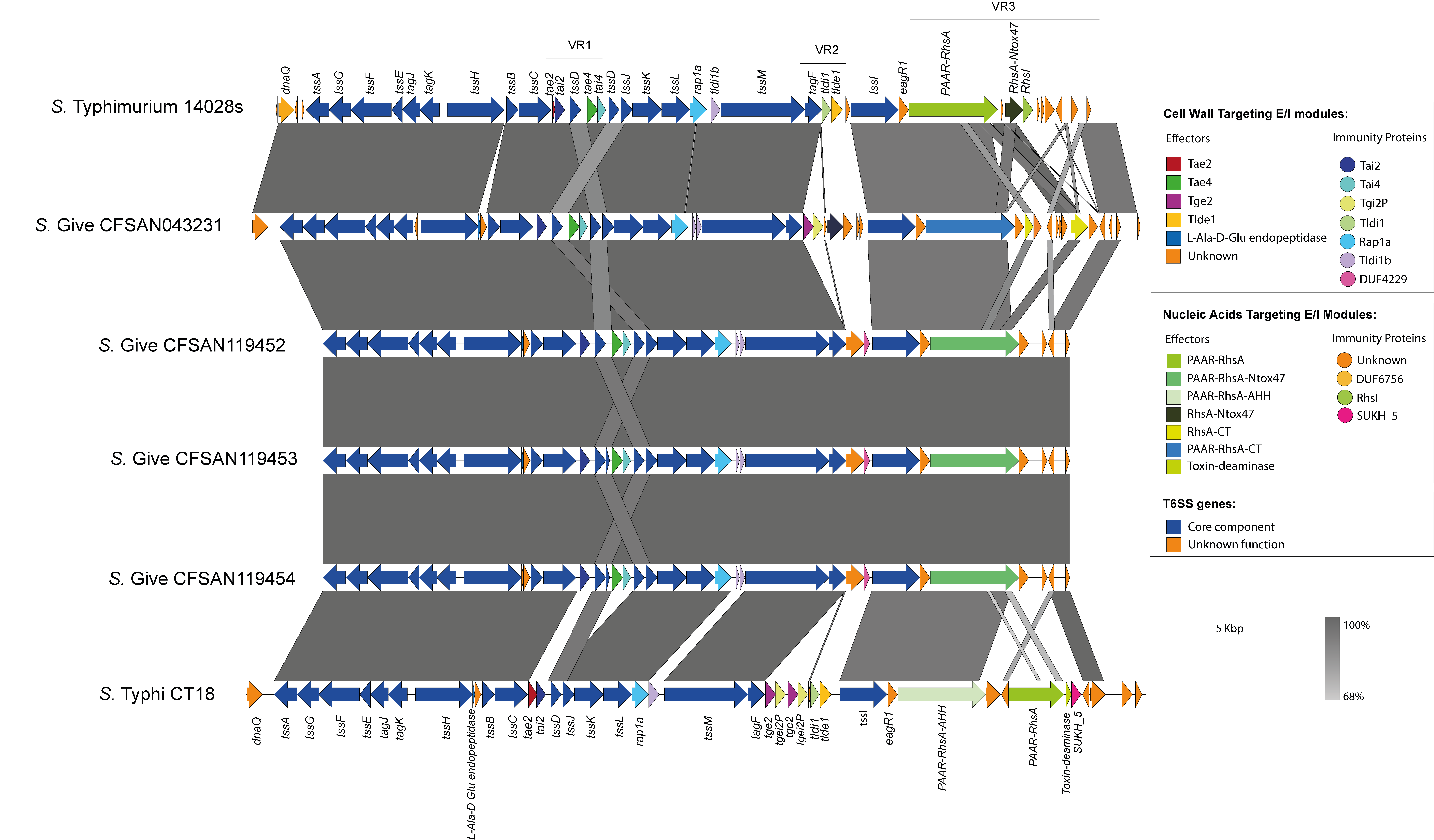

Supplement: SUPPLEMENTARY FIGURE S3 — The genetic structure and repertoire of effector proteins encoded in the SPI-6 T6SS gene cluster vary among isolates of serotype S. Give. Comparative genomic analysis of the SPI-6 T6SS cluster in isolates of S. Give. BLASTn sequence alignment was performed and visualized using EasyFig version 2.2.5 (Sullivan et al., 2011). ORFs encoding E/I modules are highlighted in different colors according to the confirmed or predicted functions. SPI-6 T6SS gene clusters from S. Typhimurium 14028s and S. Typhi CT18 were used for comparative purposes. [file Image_3.TIF]

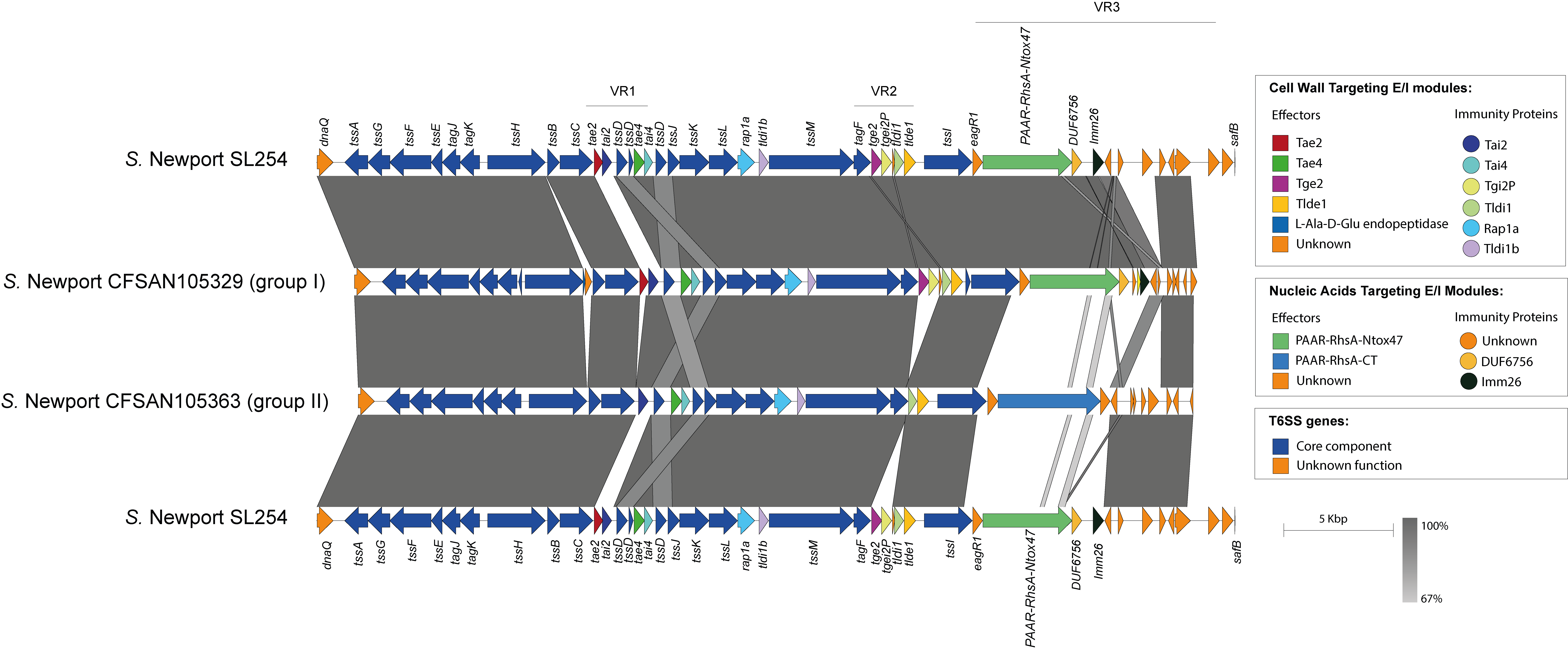

Supplement: SUPPLEMENTARY FIGURE S4 — The genetic structure and repertoire of effector proteins encoded in the SPI-6 T6SS gene cluster vary among isolates of serotype S. Newport. Comparative genomic analysis of the SPI-6 T6SS cluster in isolates of S. Newport. BLASTn sequence alignment was performed and visualized using EasyFig version 2.2.5 (Sullivan et al., 2011). ORFs encoding E/I modules are highlighted in different colors according to the confirmed or predicted functions. SPI-6 T6SS gene cluster from S. Newport SL254 was used for comparative purposes. [file Image_4.TIF]

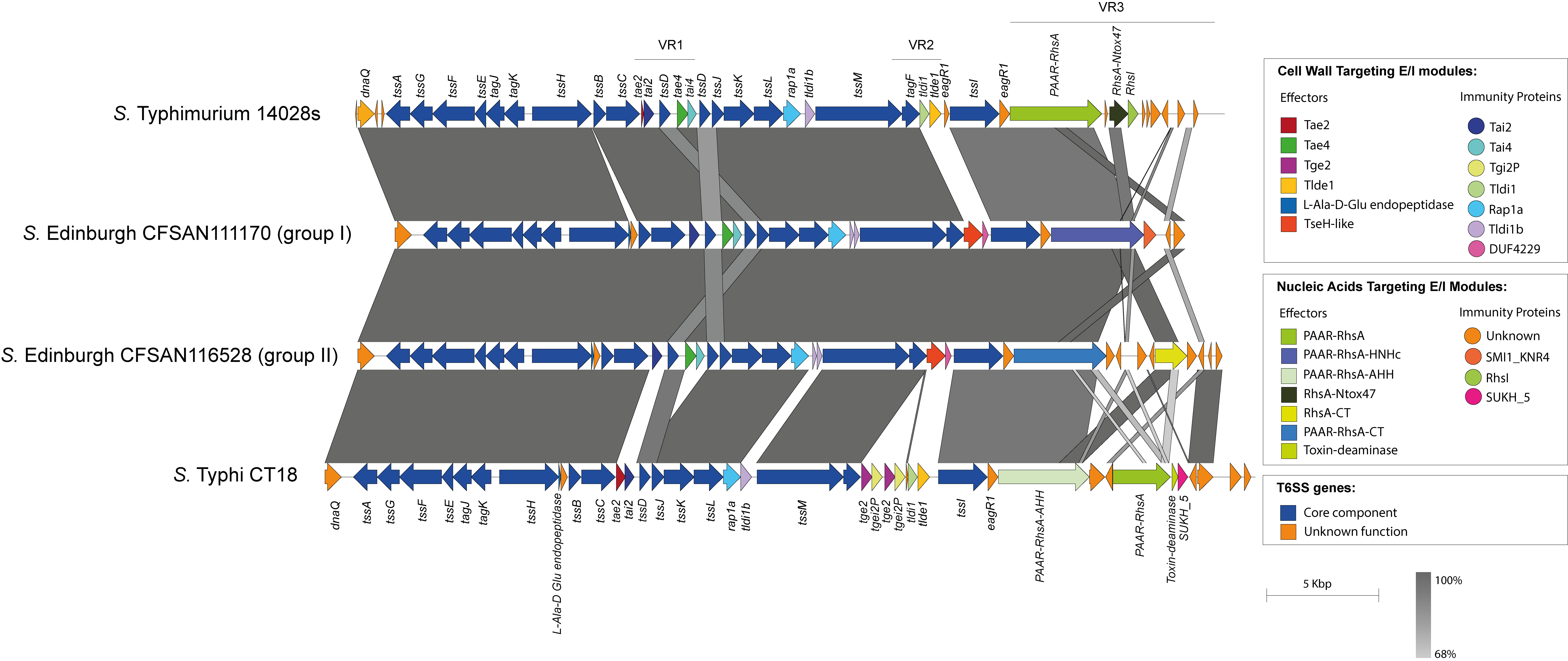

Supplement: SUPPLEMENTARY FIGURE S5 — The genetic structure and repertoire of effector proteins encoded in the SPI-6 T6SS gene cluster vary among isolates of serotype S. Edinburgh. Comparative genomic analysis of the SPI-6 T6SS cluster in isolates of S. Edinburgh. BLASTn sequence alignment was performed and visualized using EasyFig version 2.2.5 (Sullivan et al., 2011). ORFs encoding E/I modules are highlighted in different colors according to the confirmed or predicted functions. SPI-6 T6SS gene clusters from S. Typhimurium 14028s and S. Typhi CT18 were used for comparative purposes. [file Image_5.TIF]

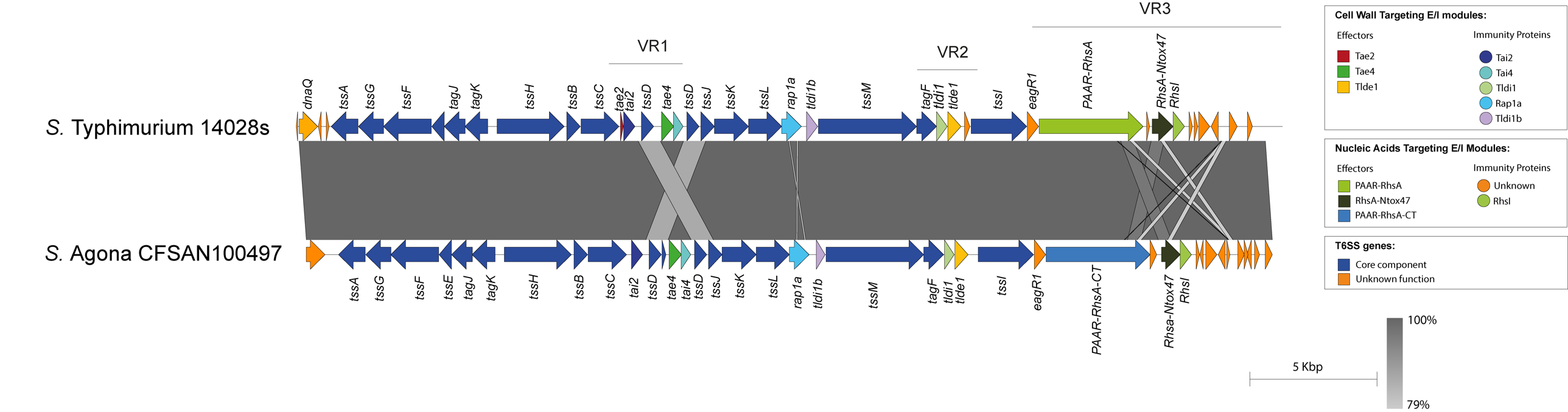

Supplement: SUPPLEMENTARY FIGURE S6 — The SPI-6 T6SS gene cluster from S. Agona CFSAN100497 and S. Typhimurium 14028s share high sequence identity. Comparative genomic analysis of the SPI-6 T6SS gene cluster of S. Agona CFSAN100497 and S. Typhimurium 14028s. BLASTn sequence alignment was performed and visualized using EasyFig version 2.2.5 (Sullivan et al., 2011). ORFs encoding E/I modules are highlighted in different colors according to the confirmed or predicted functions. [file Image_6.TIF]
